# A positive feedback loop between RIP3 and JNK controls non-alcoholic steatohepatitis

**DOI:** 10.15252/emmm.201403856

**Published:** 2014-06-24

**Authors:** Jérémie Gautheron, Mihael Vucur, Florian Reisinger, David Vargas Cardenas, Christoph Roderburg, Christiane Koppe, Karina Kreggenwinkel, Anne Theres Schneider, Matthias Bartneck, Ulf Peter Neumann, Ali Canbay, Helen Louise Reeves, Mark Luedde, Frank Tacke, Christian Trautwein, Mathias Heikenwalder, Tom Luedde

**Affiliations:** 1 Department of Gastroenterology, Digestive Diseases and Intensive Care Medicine (Department of Medicine III), University Hospital RWTH AachenAachen, Germany; 2 Interdisciplinary Centre for Clinical Research Aachen, University Hospital RWTH AachenAachen, Germany; 3 Institute of Virology, Technische Universität München and Helmholtz Zentrum München für Gesundheit und Umwelt (HMGU)Munich, Germany; 4 Department of Visceral and Transplantation Surgery, University Hospital RWTH AachenAachen, Germany; 5 Department of Gastroenterology and Hepatology, University Hospital, University Duisburg-EssenEssen, Germany; 6 The Liver Group, Department of Medicine, Freeman Hospital, Newcastle-upon-Tyne Hospitals NHS Foundation TrustNewcastle-upon-Tyne, UK; 7 Department of Cardiology and Angiology, University Hospital KielKiel, Germany

**Keywords:** biliary ductular reaction, Caspase-8, liver fibrosis, MCP-1, necroptosis

## Abstract

Non-alcoholic fatty liver disease (NAFLD) represents the most common liver disease in Western countries and often progresses to non-alcoholic steatohepatitis (NASH) leading ultimately to liver fibrosis and liver cancer. The occurrence of hepatocyte cell death—so far characterized as hepatocyte apoptosis—represents a fundamental step from benign steatosis toward progressive steatohepatitis. In contrast, the function of RIP3-dependent “necroptosis” in NASH and NASH-induced fibrosis is currently unknown. We show that RIP3 is upregulated in human NASH and in a dietary mouse model of steatohepatitis. RIP3 mediates liver injury, inflammation, induction of hepatic progenitor cells/activated cholangiocytes, and liver fibrosis through a pathway suppressed by Caspase-8. This function of RIP3 is mediated by a positive feedback loop involving activation of Jun-(N)-terminal Kinase (JNK). Furthermore, RIP3-dependent JNK activation promotes the release of pro-inflammatory mediators like MCP-1, thereby attracting macrophages to the injured liver and further augmenting RIP3-dependent signaling, cell death, and liver fibrosis. Thus, RIP3-dependent necroptosis controls NASH-induced liver fibrosis. This pathway might represent a novel and specific target for pharmacological strategies in patients with NASH.

**Subject Categories** Digestive System; Metabolism

## Introduction

Non-alcoholic fatty liver disease (NAFLD) is the most common chronic liver disease in the Western world (Vernon *et al*, 2011). The term non-alcoholic steatohepatitis (NASH) defines a more aggressive disease entity within the spectrum of NAFLD that is often associated with obesity, type 2 diabetes, and the metabolic syndrome (Schattenberg & Schuppan, 2011). In NASH, advanced fibrosis and cirrhosis are primary determinants of an increased overall and liver-related mortality (Schuppan & Afdhal, 2008; Bhala *et al*, 2011; Poelstra & Schuppan, 2011), underlining that pharmacological inhibition of liver fibrogenesis or induction of fibrosis regression is a fundamental goal in this disease (Schattenberg & Schuppan, 2011). Despite several molecular targets that were addressed in NASH patients in recent clinical trials (Schuppan & Kim, 2013), no effective pharmacological strategy against NASH-induced liver fibrosis has yet entered clinical practice, highlighting the need to identify novel-signaling pathways regulating the transition from NASH to hepatic fibrosis.

One fundamental difference between benign steatosis and progressive steatohepatitis is the occurrence of massive hepatocyte cell death, at present classified as hepatocyte apoptosis (Wree *et al*, 2013). Apoptosis can be triggered by ligation of death receptors like tumor necrosis factor (TNF) receptor by their cognate ligands and represents a highly synchronized procedure depending on activation of aspartate-specific proteases known as caspases (Chakraborty *et al*, 2012). Of these, Caspase-8 represents a key upstream caspase that engages to the death-inducing signaling complex (DISC) via the adaptor molecule FADD (Chakraborty *et al*, 2012). NASH is histologically characterized by hepatocyte apoptosis and varying degrees of fibrosis in the setting of hepatocyte lipid accumulation (Schattenberg & Schuppan, 2011). In line with this observation, previous functional studies in animal models and clinical studies have focused on the potential role of apoptosis in NASH development (Witek *et al*, 2009; Anstee *et al*, 2010; Ratziu *et al*, 2012; Hatting *et al*, 2013). However, necrosis and necro-inflammation are also histological characteristics of human NASH (Malhi & Gores, 2008; Schattenberg & Schuppan, 2011), suggesting that alternative cell-death forms might play a role in the pathogenesis of this disease. It was recently discovered that necroptosis—programmed necrosis depending on the kinases RIP1 and RIP3—represents an alternative programmed cell-death pathway downstream of the TNF receptor (Cho *et al*, 2009; He *et al*, 2009; Zhang *et al*, 2009). RIP3 mediates necroptosis through activation of mixed lineage kinase domain-like protein (MLKL) (Sun *et al*, 2012). Necroptosis plays a role in the regulation of chronic inflammation in the pancreas, gut, and skin (He *et al*, 2009; Bonnet *et al*, 2011; Welz *et al*, 2011). Moreover, necroptosis is activated in patients with alcoholic liver injury (Roychowdhury *et al*, 2013), but the role of RIP3 in NASH is currently unknown.

To examine the functional role of RIP3 in NASH development, we applied the methionine- and choline-deficient (MCD) diet-induced model of steatohepatitis that mimics important features of human NASH, including the development of steatohepatitis, CYP2E1 overexpression, and increased lipid peroxidation as well as the promotion of NASH toward hepatic fibrosis (Schattenberg *et al*, 2006). In addition, most previous data on the relation between cell death and NASH were gained in this respective model (Csak *et al*, 2011; Hatting *et al*, 2013). We show that RIP3 controls NASH development in a Caspase-8-dependent manner by a pathway involving activation of Jun-(N)-terminal kinase and thus might represent a promising target for future therapeutic strategies in patients with chronic metabolic liver disease.

## Results

### RIP3 mediates liver injury in MCD-diet-induced NASH

**Figure 1:**
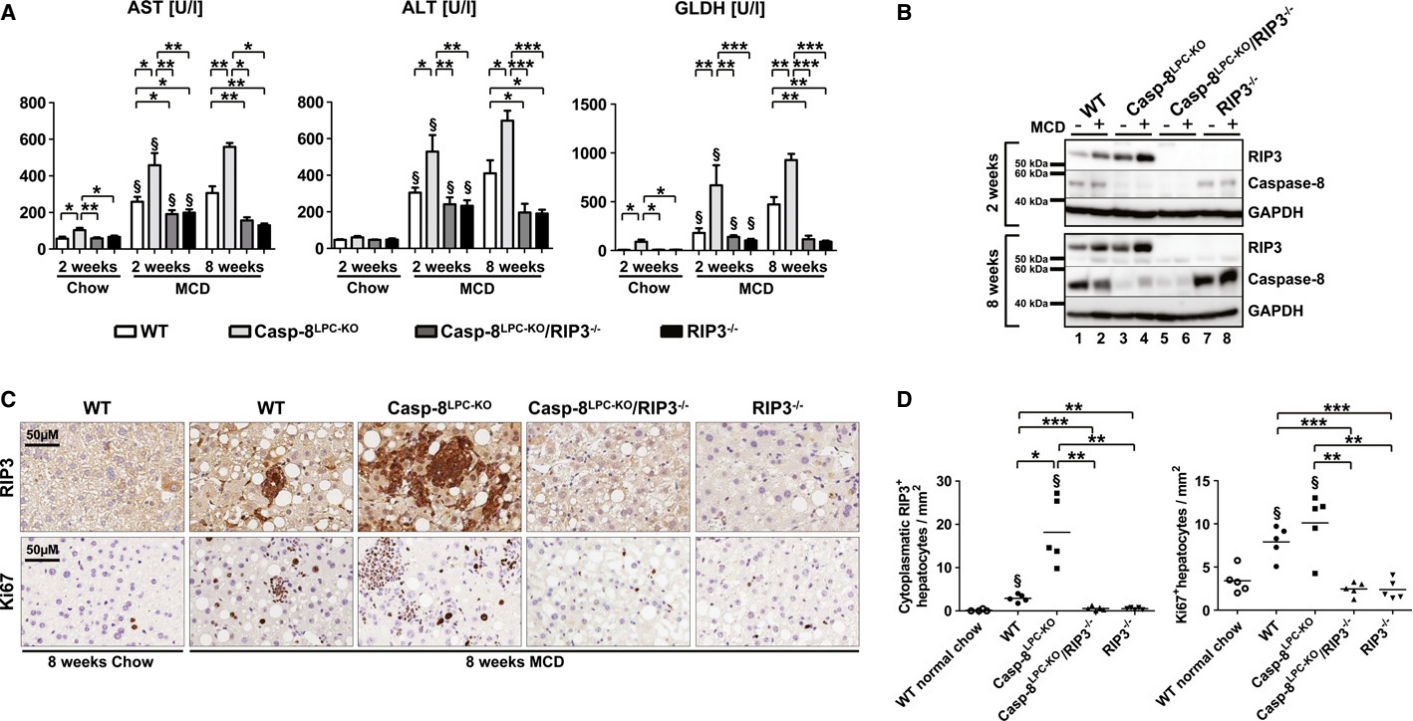
RIP3 is induced in murine livers following MCD-diet feeding and promotes hepatic injury in Caspase-8-deficient livers A   Analysis of serum levels of AST, ALT, and GLDH after 2 and 8 weeks of MCD-diet or 2 weeks of normal chow. Results are shown as mean ± SEM, *n* = 6 (2 weeks normal chow) *n* = 12 (2 weeks MCD), and *n* = 5 (8 weeks MCD). ^§^indicates that serum levels are significantly increased from basal level. B   Western blots analysis on liver extracts from MCD-diet-fed (2- and 8-weeks) animals and control mice with antibodies against RIP3, Caspase-8, and GAPDH as a loading control. C   Immunohistochemical (RIP3, Ki67) analysis on representative liver sections from the indicated mice fed for 8 weeks with MCD-diet. D   Statistical analysis of RIP3^+^ and Ki67^+^ hepatocytes. Results are shown as mean, *n* = 5. ^§^indicates that RIP3^+^ and Ki67^+^ cells are significantly increased from basal WT group. Data information: The exact *P*-values of each experiment and specific tests used are provided in the Supplementary Table S1. Source data is available online for this figure.

In order to examine the differential functions of RIP3-dependent necroptosis versus Caspase-8-dependent apoptosis in NASH, we generated mice with conditional deletion of *Caspase-8* in liver parenchymal cells (LPC)—hepatocytes and cholangiocytes—(Casp-8^LPC-KO^), constitutive ablation of *Rip3* in all cells (RIP3^−/−^), and mice with combined conditional and constitutive deletions of Caspase-8 and RIP3, respectively (Casp-8^LPC-KO^/RIP3^−/−^) (Supplementary Fig S1). These groups of mice were treated for either 2 or 8 weeks with MCD-diet or normal chow as control and were first analyzed for the degree of liver injury. As described previously (Vucur *et al*, 2013), Casp-8^LPC-KO^ mice showed a moderate increase in serum levels of aspartate aminotransferase (AST) and glutamate dehydrogenase (GLDH) but not alanine aminotransferase (ALT) in mice fed with normal chow (Fig 1A). After 2 and 8 weeks of MCD-diet, serum levels of AST, ALT, and GLDH were increased in all groups compared to the respective control animals on normal chow (Fig 1A). Strikingly, enzyme levels were more increased in Casp-8^LPC-KO^ animals at both time points compared to all other experimental groups (Fig 1A). In contrast, after 8 weeks of MCD-diet, all liver enzymes were reduced in RIP3^−/−^ mice and Casp-8^LPC-KO^/RIP3^−/−^ animals compared to WT mice (Fig 1A). Together, these findings indicate that RIP3 mediates liver injury upon MCD-diet feeding in mice.

Based on this observation, we further examined intrahepatic expression levels of RIP3 in this model. It was previously shown that necroptotic cell death in the liver or pancreas is associated with an increase in RIP3 protein levels (He *et al*, 2009; Vucur *et al*, 2013). In line, 2 weeks of MCD-diet feeding led to a strong induction of RIP3 protein levels in Western blot analysis (Fig 1B). Of note, this induction of RIP3 expression was even augmented in Casp-8^LPC-KO^ animals with abrogated Caspase-8-expression (Fig 1B), while—as expected—no RIP3 expression was detected in Casp-8^LPC-KO^/RIP3^−/−^ and RIP3^−/−^ livers (Fig 1B). Immunohistological analyses of livers of mice after 8 weeks of MCD-diet feeding confirmed high expression levels of RIP3 in WT mice and even stronger expression in Casp-8^LPC-KO^ livers (Fig 1C and D). In contrast, immunohistological staining for cleaved Caspase-3 revealed similarly low levels of cleaved Casp-3^+^ hepatocytes in all groups (Supplementary Fig S2). The fact that cleavage of Caspase-3 was also detected in mouse livers with conditional deletion of Caspase-8 indicated that apoptosis in this model can be activated by Caspase-8-independent signaling cascades, e.g. via the mitochondrial pathway (Estaquier *et al*, 2012). In line with this finding, it was previously shown that in NASH, fatty acid accumulation enhances β-oxidation and mitochondrial electron overflow, thus triggering cell death (Seifert *et al*, 2010). Finally, compensatory proliferation of parenchymal liver cells measured by staining for Ki67 correlated with overexpression of RIP3 and was most prominent in Casp-8^LPC-KO^ mice, while Casp-8^LPC-KO^/RIP3^−/−^ and RIP3^−/−^ mice showed significantly less proliferating cells than MCD-diet-fed WT mice (Fig 1C and D). Together, these observations demonstrate that activation of RIP3 represents a fundamental step in the MCD-diet NASH model mediating liver injury. Moreover, a crucial function of Caspase-8 in this model is to counterbalance RIP3-dependent liver injury.

### RIP3 controls the transition from NASH to liver fibrosis in a Caspase-8 dependent manner

**Figure 2:**
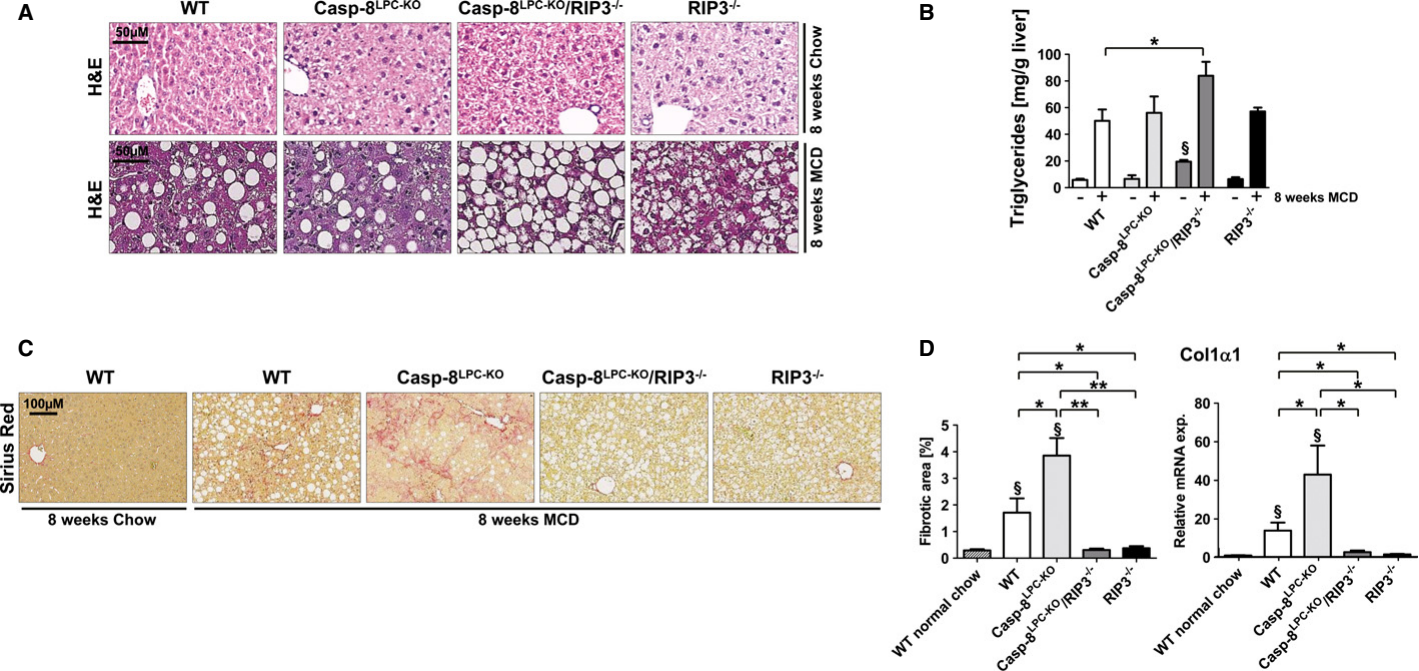
RIP3-dependent necroptosis promotes NASH-induced liver fibrosis and inflammation A   Representative H&E staining of liver slides from 16-week-old WT, Casp-8^LPC-KO^, Casp-8^LPC-KO^/RIP3^−/−^, and RIP3^−/−^ mice fed for 8 weeks with normal chow (upper panel) or MCD-diet (lower panel). B   Intrahepatic triglycerides levels in WT, Casp-8^LPC-KO^, Casp-8^LPC-KO^/RIP3^−/−^, and RIP3^−/−^ fed for 8 weeks with MCD-diet or control chow, results are shown as mean ± SEM, *n* = 5 per group. ^§^indicates that triglycerides are significantly increased from Casp-8^LPC-KO^/RIP3^−/−^ to the others groups of mice fed with normal chow. C   Representative Sirius Red stainings of liver slides from 8-week-old female WT, Casp-8^LPC-KO^, Casp-8^LPC-KO^/RIP3^−/−^, and RIP3^−/−^ mice fed for 8 weeks with MCD-diet. D   Left: statistical quantification of light polarized Sirius Red pictures, results are shown as mean, *n* = 5 per group. Right: *Col1*α*1* mRNA levels in these livers were determined by qRT-PCR. Values were calculated relative to WT mice fed with normal show, and β-catenin was used as an internal standard, *n* = 5 per group. ^§^indicates that values are significantly increased from basal level. Error bars represent SEM. Data information: The exact *P*-values of each experiment and specific tests used are provided in the Supplementary Table S1.

Based on the differential functions of RIP3 and Caspase-8 in controlling liver injury in response to MCD-diet feeding, we next tested their influence on the pathogenesis of steatosis and NASH-induced liver fibrosis. Hematoxylin and eosin (H&E) staining revealed minimal steatosis in all groups of mice after 8 weeks of normal chow feeding (Fig 2A). Of note, mice with combined deletions of *Caspase-8* and *Rip3* (Casp-8^LPC-KO^/RIP3^−/−^ mice) on normal chow already displayed increased triglyceride (TG) levels compared to the other experimental groups (Fig 2B). Eight weeks of MCD-diet feeding triggered hepatic fat accumulation above control levels in all groups (Fig 2A and B). In line with the control groups, MCD-diet feeding led to significantly higher intrahepatic TG contents in Casp-8^LPC-KO^/RIP3^−/−^ mice compared to all other experimental groups, suggesting that inactivation of both programmed cell-death pathways augments hepatic fat accumulation in the MCD-diet model.

The presence of apoptosis in NAFLD patients has been taken as a predictor to develop progressive fibrosis (Witek *et al*, 2009; Anstee *et al*, 2010; Ratziu *et al*, 2012). In contrast, while necrosis is also found as histological characteristic in human NASH (Malhi & Gores, 2008; Schattenberg & Schuppan, 2011), the functional relation to fibrosis is presently poorly defined. We therefore investigated the correlation between the occurrence of liver fibrosis and the activation of necroptosis in the MCD-diet model. Sirius red staining and qRT-PCR analysis for Collagen-1α1 expression after 2 weeks (Supplementary Fig S3) and 8 weeks of MCD-diet feeding (Fig 2C and D) revealed no significant liver fibrosis at the early time points and moderate fibrosis at the later time point in WT animals. In contrast, correlating with induction of RIP3 expression levels, Casp-8^LPC-KO^ animals displayed strongly increased intrahepatic fibrosis at both time points, whereas hepatic fibrogenesis was strongly reduced in RIP3^−/−^ single-mutant mice and Casp-8^LPC-KO^/RIP3^−/−^ double-mutant animals compared to WT and Casp-8^LPC-KO^ mice (Fig 2C and D; Supplementary Fig S3). These data indicate that RIP3-dependent necroptosis promotes NASH-induced liver fibrosis. Moreover, activation of Caspase-8 inhibits RIP3-dependent liver fibrosis in NASH.

We have further addressed the question whether the previously shown pro-fibrogenic effect of RIP3 is specific for liver fibrosis in response to hepatic steatosis or represents a general principle in hepatic fibrogenesis. To test this, we used an alternative, very well-established model of experimental liver fibrosis relying on repetitive injections of the substance CCl_4_ into mice and applied this model for 2 and 6 weeks to WT, Casp-8^LPC-KO^, Casp-8^LPC-KO^/RIP3^−/−^, and RIP3^−/−^ mice. This treatment led to the development of areas of parenchymal cell necrosis in Casp-8^LPC-KO^ mice (Supplementary Fig S4). However, it did not result in a significantly increased degree of fibrosis between the groups of mice in quantitative analysis of Sirius Red staining (Supplementary Fig S4), supporting the hypothesis that RIP3 might represent a specific target in fatty liver-related liver fibrosis.

### RIP3-activation in NASH promotes inflammation and hepatic recruitment of monocytes/macrophages

**Figure 3:**
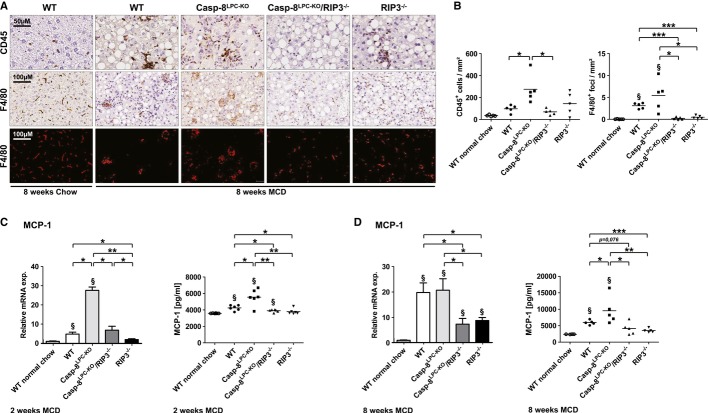
RIP3-dependent necroptosis promotes NASH-induced liver fibrosis through MCP-1 release A   Immunohistochemical analysis of CD45 (upper panel) and F4/80 (middle panel) on representative liver sections from the indicated mice fed for 8-weeks with MCD-diet or normal chow. The lower panel shows deconvoluted pictures from the F4/80 stains. B   Statistical analysis of CD45^+^ and F4/80^+^ cells. Results are shown as mean, *n* = 5. ^§^indicates that F4/80^+^ foci are significantly increased from basal WT group. C   Left: *MCP-1* mRNA levels were assessed by RT-PCR after 2 weeks of MCD-diet feeding. Values were calculated relative to WT mice fed with normal chow, and β-catenin was used as an internal standard, *n* = 6 per group. Right: FACS-based microbeads fluorescence assay for MCP-1 expression in liver protein homogenates. Results are shown as mean, *n* = 6 per group. § shows that values are significantly increased from basal level. Error bars indicate SEM. D   Left: *MCP-1* mRNA levels were assessed by RT-PCR after 8 weeks of MCD-diet feeding. Values were calculated relative to WT mice fed with normal chow, and β-catenin was used as an internal standard, *n* = 5 per group. ^§^indicates that values are significantly increased from basal level. Error bars represent SEM. Right: FACS-based microbeads fluorescence assay for MCP-1 expression in liver protein homogenates. Results are shown as mean, *n* = 5 per group. ^§^shows that values are significantly increased from basal level. Error bars indicate SEM. Data information: The exact *P*-values of each experiment and specific tests used are provided in the Supplementary Table S1.

Inflammation represents a fundamental factor linking liver injury with hepatic fibrosis (Tacke *et al*, 2008; Schuppan & Kim, 2013). We therefore examined the association between liver fibrosis and inflammation in response to necroptosis upon MCD-diet feeding. As assessed by immunohistochemistry, 8 weeks of MCD-diet feeding increased the number of infiltrating CD45^+^ immune cells in all experimental groups of mice compared to WT mice fed with normal chow. Of note, inflammation was even higher in Casp-8^LPC-KO^ mice and correlated with the strong expression of RIP3 as the number of CD45^+^ immune cells was reduced to WT levels in Casp-8^LPC-KO^/RIP3^−/−^ mice and RIP3^−/−^ animals (Fig 3A and B). We next investigated the impact of infiltrating monocytes, which play a pro-fibrogenic role in different experimental models of liver fibrosis as well as human liver disease (Zimmermann & Tacke, 2011). As demonstrated by immunohistochemistry, 8 weeks of MCD-diet feeding significantly increased the number of inflammatory foci containing monocytes in WT mice and even more in Casp-8^LPC-KO^ livers (Fig 3A and B). In contrast, the emergence of these foci was completely abrogated by the deletion of *Rip3* as seen in Casp-8^LPC-KO^/RIP3^−/−^ and RIP3^−/−^ animals (Fig 3A and B). The activation role of RIP3 in the initiation of inflammation was confirmed by FACS analyses in livers of mice fed for 2 weeks with MCD-diet, revealing significantly lower numbers of F4/80^+^ cells in Casp-8^LPC-KO^/RIP3^−/−^ mice and RIP3^−/−^ animals than seen in Casp-8^LPC-KO^ mice (Supplementary Fig S5). Taken together, these results indicate that RIP3-dependent inflammation and recruitment of monocytes represents an important mechanism for promoting hepatic fibrosis in mice fed with MCD-diet.

We further tested which inflammatory mediators might be involved in linking necroptosis of parenchymal liver cells with monocyte recruitment and increased hepatic fibrosis. Interestingly, while many inflammatory cytokines and chemokines such as Interleukin (IL)-1α, IL-1β, IL-6, CCL1 and CCL8, and CCL17 did not show a clearly distinct regulation between WT and Casp-8^LPC-KO^ mice fed for 2 weeks with MCD-diet on mRNA level (Supplementary Fig S6), qRT-PCR analysis and FACS-based microbeads fluorescence assay on liver extracts from the different experimental groups revealed a strong increase in intrahepatic levels of MCP-1 (CCL2) in Casp-8^LPC-KO^ mice compared to WT mice at that time point (Fig 3C). In contrast, MCP-1-levels were markedly reduced in Casp-8^LPC-KO^/RIP3^−/−^, and RIP3^−/−^ mice (Fig 3C), which was confirmed after 8 weeks of MCD-diet feeding (Fig 3D). Given previous reports on the essential functional role of the MCP-1/CCR2 axis in monocyte recruitment and liver fibrosis (Seki *et al*, 2009; Baeck *et al*, 2012), these data indicate that MCP-1 represents one important factor linking RIP3-dependent necroptosis in NASH with liver fibrosis. Further analyses revealed increased TNF levels in WT and Casp-8^LPC-KO^ mice after 8 weeks of MCD feeding, as well as a strong correlation between RIP3 expression levels and levels of TGF-β2 (Supplementary Fig S7), which is in line with a recent report showing a prominent role of TGF-β in the regulation of NASH-associated hepatocyte cell death (Yang *et al*, 2014).

The *alfp*-cre line used to generate Casp-8^LPC-KO^ animals mediates genetic excision exclusively in liver parenchymal cells (LPC) (Kellendonk *et al*, 2000), arguing for a specific function of RIP3 in this respective cell compartment in driving liver injury and subsequent fibrogenesis in Casp-8^LPC-KO^ mice upon MCD-diet feeding. However, in order to exclude that constitutive deletion of *Rip3* in Casp-8^LPC-KO^/RIP3^−/−^ mice resulted in general signaling defects of immune cells as a reason for the rescue of these double-mutant animals from hepatic fibrosis, we isolated and cultured monocytes from murine bone marrow of WT, Casp-8^LPC-KO^, Casp-8^LPC-KO^/RIP3^−/−^, and RIP3^−/−^ mice. Stimulation with lipopolysaccharide (LPS) resulted in very similar patterns of chemokine and cytokine expression (Supplementary Fig S8), suggesting that the functional activity of macrophages is not abrogated in RIP3^−/−^ and Casp-8^LPC-KO^/RIP3^−/−^ mice.

### RIP3-expression in murine and human NASH livers

**Figure 4:**
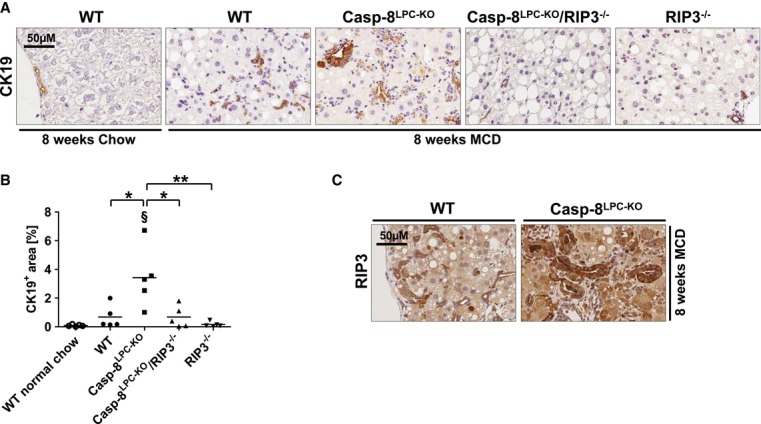
RIP3-dependent necroptosis mediates expansion of progenitor cells that expressed high levels of RIP3 A   Immunohistochemical (CK19) analysis on representative liver sections from the indicated mice fed for 8-weeks with MCD-diet. B   Statistical analysis of CK19^+^ cells. Results are shown as mean, *n* = 5. ^§^shows that CK19^+^ cells are significantly increased from basal WT group. C   Immunohistochemical (RIP3) analysis on representative liver sections from WT and Casp-8^LPC^^-^^KO^ mice fed for 8 weeks with MCD-diet. Data information: The exact *P*-values of each experiment and specific tests used are provided in the Supplementary Table S1.

Most liver diseases and also NASH—once advanced—develop into a portal fibrosis with proliferation of biliary progenitors and activated cholangiocytes, which typically form small clusters and non-functional biliary structures (Richardson *et al*, 2007; Schuppan & Kim, 2013). We therefore tested the relation between RIP3-dependent necroptosis and a biliary ductular reaction in the MCD-diet model. Strikingly, immunostaining for Cytokeratin (CK)-19 revealed a strong expansion of clusters of biliary cells / progenitor cells in livers of Casp-8^LPC-KO^ mice, which was not seen in combined or single RIP3-mutants (Fig 4A and B). Of note, immunostaining for RIP3 and specific analysis in areas of ductular reactions revealed that many cholangiocytes expressed even higher levels of RIP3 than the surrounding hepatocytes (Fig 4C).

**Figure 5:**
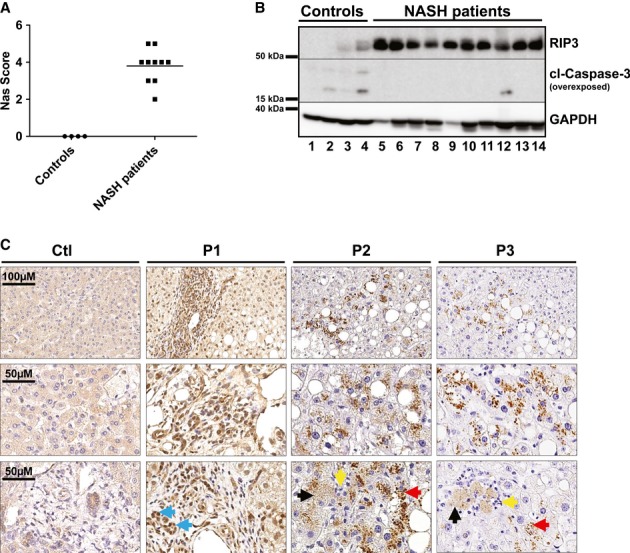
RIP3 is overexpressed in livers of human NASH patients A   Western blot analysis of RIP3 in control livers (*n* = 4) and human NASH patients (*n* = 10), using antibodies against RIP3, cleaved Caspase-3, and GAPDH as a loading control. B   NAS scores of control livers and human NASH samples, corresponding to the Western blot analysis for RIP3. C   Immunostaining analyses of RIP3 in control livers and human NASH patients (P1, P2, P3) (blue arrows indicate progenitor/biliary cells, black arrows indicate necrotic hepatocytes, red arrows show clusters of RIP3 in hepatocytes, and yellow arrows indicate inflammatory cells grouped around dying hepatocytes). Pictures are representative for 27 samples examined. Source data are available online for this figure.

It was previously demonstrated in liver samples from human NASH patients that RIP3 is strongly upregulated on RNA level to more than 40-fold compared to healthy controls (Csak *et al*, 2011). In order to provide further evidence for a function of RIP3 in human NASH, we examined RIP3 expression in livers of NASH patients (as demonstrated histologically by increased NAS score, see Fig 5A) by Western blot and immunohistochemistry. On protein levels, RIP3 was strongly upregulated in NASH patients compared to controls (Fig. 5B). Immunostaining of NASH patient livers revealed strong RIP3 expression in hepatocytes, often neighboring areas of fat deposition (Fig 5C). Of note, RIP3 often showed a granule-like staining pattern (Fig 5C), similar to previous imaging results in MEF cells with activated RIP3 signaling depicting clustering of RIP1/RIP3 (Li *et al*, 2012). Finally, RIP3 was often overexpressed in cells morphologically reflecting cholangiocytes / bile duct cells (Fig 5C), similar to our previous findings in mouse livers. These findings support the hypothesis that also in human NASH, liver cells are sensitized to necroptotic cell death. Moreover, in murine as well as human NASH, biliary cells express high levels of RIP3, pointing toward cell-type specific functions of this pathway in the liver.

### A positive feedback loop involving activation of Jun-(N)-terminal Kinase (JNK) mediates RIP3-dependent inflammation and hepatic fibrosis upon MCD feeding

**Figure 6:**
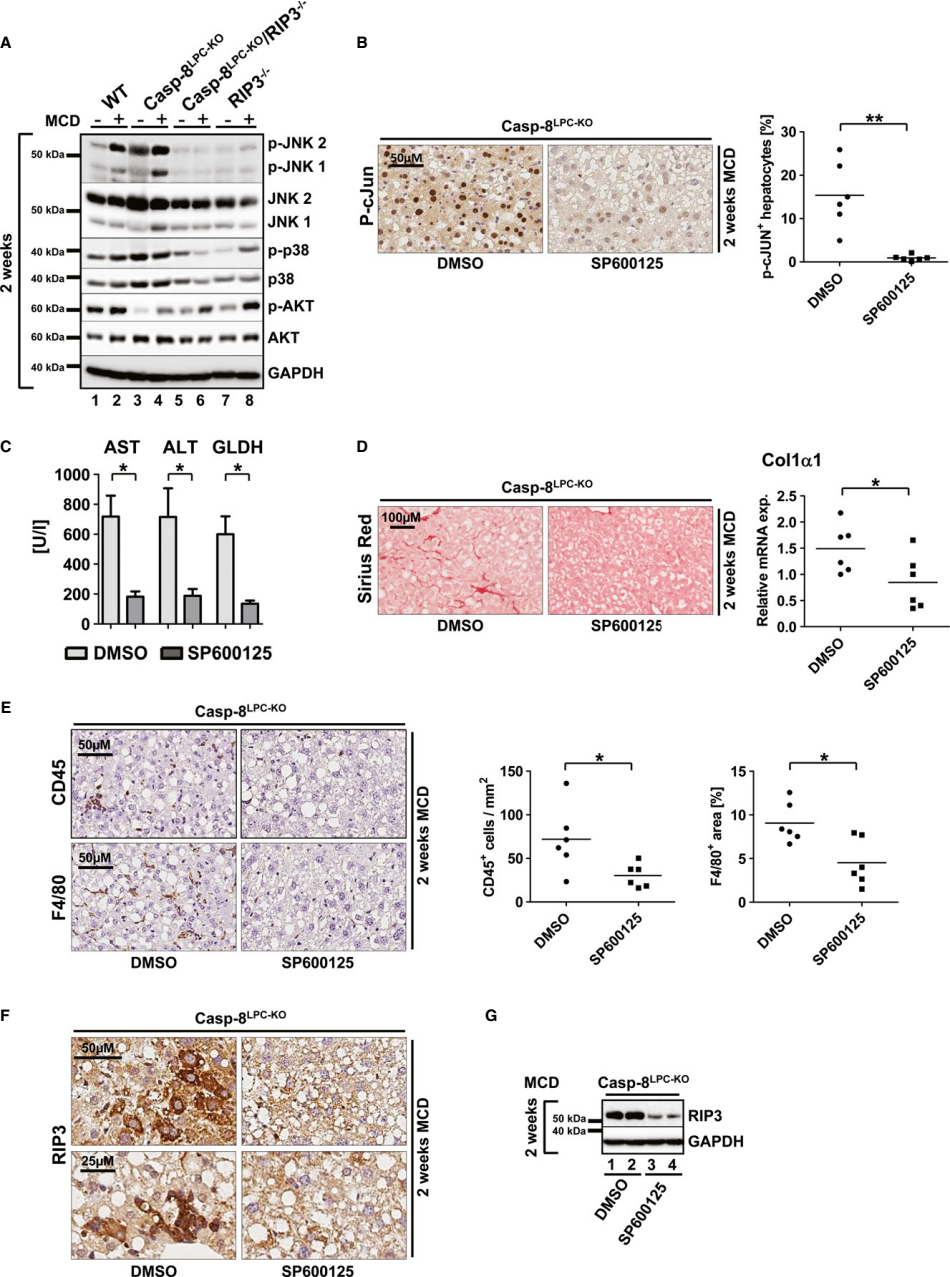
RIP3 mediates MCD-diet-induced NASH and liver fibrosis through activation of Jun-(N)-terminal kinase (JNK) A   Western blot analysis of whole liver protein extracts from WT, Casp-8^LPC-KO^, Casp-8^LPC-KO^/RIP3^−/−^, and RIP3^−/−^ mice fed with MCD-diet for 14 days, using antibodies against the phosphorylated and active forms of JNK, AKT, p38 and GAPDH, JNK, AKT, and p38 as loading controls. B   Immunohistochemical (left) and statistical analysis (right) of nuclear p-c-Jun^+^ hepatocytes on representative liver sections from Casp-8^LPC-KO^ mice treated with SP600125 or vehicle (DMSO) for 2 weeks under MCD-diet, *n* = 6 per group. C   Analysis of serum levels of AST, ALT, and GLDH of vehicle-treated and SP600125-treated mice after 2 weeks of MCD-diet feeding. Results are shown as mean ± SEM, *n* = 6 per group. D   Left: representative Sirius Red stainings of mice treated with vehicle substance (DMSO) or SP600125 for 2 weeks. Right: *Col1*α*1* mRNA levels quantification by qRT-PCR, *n* = 6 per group. Error bars represent SEM. E   Immunohistochemical (left) and statistical analysis (right) of CD45^+^ and F4/80^+^ cells on representative liver sections from Casp-8^LPC^^-^^KO^ mice treated with SP600125 or vehicle (DMSO) for 2 weeks with MCD-diet, *n* = 6 per group. F   Immunostaining analysis of RIP3 in Casp-8^LPC^^-^^KO^ mice treated with SP600125 or vehicle (DMSO) for 2 weeks with MCD-diet. G   Western blot analysis of RIP3 in Casp-8^LPC^^-^^KO^ mice treated with SP600125 or vehicle (DMSO) for 2 weeks with MCD-diet. Data information: The exact *P*-values of each experiment and specific tests used are provided in the Supplementary Table S1. Source data are available online for this figure.

We finally aimed at further evaluating which potential downstream pathway mediated RIP3-dependent liver injury, inflammation, and fibrosis upon MCD feeding. To test this, we first examined the activation status of stress-related signaling cascades in the different knockout models after 2 weeks of MCD-diet feeding. As shown by Western blot analysis using phospho-specific antibodies, increased activation of RIP3 and fibrosis upon MCD-diet feeding correlated with increased phosphorylation and activation of the kinase Jun-(N)-terminal Kinase (JNK) in WT and even more in Casp-8^LPC-KO^ livers (Fig 6A), which was abolished in Casp-8^LPC-KO^/RIP3^−/−^ and RIP3^−/−^ mice (Fig 6A). In contrast, activation of AKT and p38α did not show a clear association with the activation status of RIP3 (Fig 6A).

Given previous reports on the crucial function of JNK in the mediation of NASH fibrosis (Schattenberg *et al*, 2006), we further examined the functional role of JNK in RIP3-dependent NASH fibrosis and treated groups of Casp-8^LPC-KO^ mice with repetitive injections of the well-established JNK-inhibitor SP600125 or vehicle substance as control in parallel to MCD-diet feeding. This treatment resulted in effective inhibition of phosphorylation of the JNK-target c-Jun (Fig 6B). JNK inhibition significantly ameliorated liver injury as shown by decreased levels of serum aminotransferases and GLDH in SP600125-treated mice (Fig 6C). In line, JNK inhibition ameliorated liver fibrosis as shown by Sirius Red staining and qRT-PCR analysis for expression of Collagen-Iα1 (Fig 6D). Moreover, decreased fibrosis in these livers went along with reduced intrahepatic numbers of CD45^+^ and F4/80^+^ cells (Fig 6E), correlating with reduced levels of MCP-1 (Supplementary Fig S9). Interestingly, immunostaining and Western blot analysis revealed that inhibition of JNK during MCD-diet feeding significantly reduced expression levels of RIP3 in parenchymal liver cells (Fig 6F and G). To further confirm a mutual interaction between RIP3 and JNK signaling, we used L929 cells and confirmed that these cells undergo necroptosis upon stimulation with the pan-Caspase-inhibitor zVAD (Supplementary Fig S10). Of note, additional treatment with the necroptosis inhibitor Nec-1 (Degterev *et al*, 2013) and also with SP600125 abolished zVAD-induced cell death. Moreover, JNK inhibition was associated with reduced RIP3 expression levels (Supplementary Fig S10). These data suggest that activation of JNK in LPC and probably non-parenchymal cells (NPC) further augments hepatic RIP3 signaling in terms of a positive feedback loop.

## Discussion

For years, the term apoptosis, describing a form of cell death depending on the activation of caspases, was used synonymously for programmed cell death (Najimi *et al*, 2009). Apoptotic death of hepatocytes is a common feature of non-alcoholic steatohepatitis, and it was shown that both extrinsic and intrinsic apoptotic pathways are involved in NASH-induced hepatocyte death (Feldstein *et al*, 2003; Feldstein & Gores, 2005). Apoptosis has been considered as a driving force of NASH-induced liver fibrosis (Chakraborty *et al*, 2012), as it promotes activation of hepatic stellate cells to hepatic myofibroblasts, promoting deposition of extracellular matrix and scar formation in the liver (Chakraborty *et al*, 2012). Biomarkers of apoptosis like Cytokeratin-18 are strongly increased in NASH patients and distinguish between simple steatosis and NASH (Wieckowska *et al*, 2006; Younossi *et al*, 2008). Finally, the paradigmatic concept of apoptosis in NASH fibrosis has led to translational approaches into clinical studies, testing the use of apoptosis inhibitors in NASH patients (Ratziu *et al*, 2012). Recently, however, it became evident that next to apoptosis, “necroptosis” represents an alternative programmed cell-death pathway (Han *et al*, 2011). Here, we demonstrate for the first time that RIP3-dependent necroptosis represents an important regulatory pathway driving the transition from NAFLD to NASH and subsequent liver fibrosis. Therefore, this pathway might represent a promising novel target for therapeutic strategies in NASH. Moreover, in contrast to previous assumptions (Hatting *et al*, 2013), our findings indicate that the main function of Caspase-8 in the MCD-NASH model is to counterbalance the deleterious hyperactivation of RIP3-dependent necroptosis, underlining the mutual inhibitory functions of RIP3 and Caspase-8 that were previously demonstrated, for example in embryonic development (Kaiser *et al*, 2011) and skin homeostasis (Weinlich *et al*, 2013).

The relevance of apoptosis for NASH progression is supported by several studies. As such, it was clearly demonstrated that usage of the pan-Caspase-inhibitor VX-166 significantly reduced liver injury and liver fibrosis in MCD-diet-fed db/db mice (Witek *et al*, 2009). However, pan-caspase inhibitors inhibit numerous caspases including the downstream executioner caspases. In addition, chemical pan-caspase inhibitors act also on Caspase-1, which mediates proteolytic cleavage of Interleukin molecules including IL-1β (Chang & Yang, 2000; Das *et al*, 2009). Given recent data that specific inhibition of Caspase-1 inhibits NASH fibrosis (Dixon *et al*, 2013), it is possible that targeting of this respective pathway might also contribute to the beneficial effects of pan-caspase inhibitors in NASH fibrosis. Moreover, our finding that also in *Caspase-8*-deficient livers apoptosis of hepatocytes is detected on a low level upon MCD-diet feeding suggests that Caspase-8-independent apoptosis are primarily activated in the MCD-diet model, which is supported by the fact that Casp-8^LPC-KO^/RIP3^−/−^ mice still showed significant liver injury in this respective model. Taken together, our present study together with previous findings indicate that both programmed cell-death pathways—(Caspase-8-independent) apoptosis and necroptosis—are involved in the pathogenesis of NASH and NASH-induced liver fibrosis. Of note, the degree of steatosis in our different genetically modified mouse models did not strictly correlate with the extent of liver injury upon MCD-diet feeding. Instead, blockage of both cell-death pathways (necroptosis and Caspase-8-dependent apoptosis) in Casp-8^LPC-KO^/RIP3^−/−^ mice resulted in an increase in intrahepatic fat accumulation in this model compared with WT or single-mutant animals, suggesting that absence of these two programmed cell-death pathways might increase the tolerance of hepatocytes to store lipids without undergoing cell death. Alternatively, given that multiple molecular interactions between programmed cell-death pathways and autophagy have been suggested (Pattingre *et al*, 2005; Yousefi *et al*, 2006), simultaneous inhibition of Caspase-8-dependent apoptosis and necroptosis might alter the activity of cellular pathways controlling lipolysis in hepatocytes (Liu & Czaja, 2013).

Our present findings diverge from a previous publication showing reduced liver damage in the MCD-diet model upon Caspase-8-deletion in hepatocytes (Hatting *et al*, 2013). It is important to note that in our study, we used the alfp-cre line (Kellendonk *et al*, 2000) that mediates highly efficient deletion of floxed genes at an early developmental time point in all parenchymal liver cells including cholangiocytes and progenitor cells, while in contrast, in the previous study, the albumin-cre (alb-cre) line was used (Postic *et al*, 1999). Of note, comparison of RIP3-expression levels between alfp-cre/Caspase-8Fl and alb-cre/Caspase-8Fl confirmed high RIP3 expression upon alfp-cre-mediated Caspase-8 deletion (Supplementary Fig S11), which is in line with previous reports on Caspase-8 deletion in other organs like skin (Weinlich *et al*, 2013). In contrast, we did not detect RIP3 upregulation upon albumin-cre-mediated deletion, further supporting the association between RIP3 expression levels and necroptotic liver injury.

In our experiments, we identified the stress-activated kinase JNK as a prominent mediator of RIP3-dependent liver injury and fibrogenesis in the MCD-diet model. Moreover, we show that mutual interactions exist between JNK- and RIP3-activation, as chemical inhibition of JNK not only ameliorated liver injury and fibrosis downstream of RIP3, but also led to reduced expression levels of RIP3 in liver parenchymal cells (LPC). Importantly, it is presently not clear whether the striking effect of chemical JNK inhibition in our model was mediated through JNK inhibition in hepatocytes or rather in non-parenchymal liver cells (NPLC). Previous data using conditional JNK-knockout mice in the Concanavalin-A model of hepatitis suggested that JNK activation in NPLC but not in LPC represents a major control mechanism in the regulation of hepatitis (Das *et al*, 2009). Hence, it is possible that JNK activation in LPC promotes RIP3-dependent liver injury through generation of inflammatory cytokines, which in turn augments RIP3 expression and activation in LPC. However, our experiments in L929 cells suggest that a cell-autonomous or intercellular feedback loop exists in hepatocytes between RIP3- and JNK-signaling. Further experiments with conditional JNK-knockout mice are needed to functionally clarify the cell-specific relation between RIP3 and JNK *in vivo*.

Another prominent finding in our study was the strong, RIP3-dependent induction of biliary cells in the MCD-diet model. Activated cholangiocytes are related, if not identical to biliary progenitor cells (Schuppan & Kim, 2013). These cells can proliferate in response to hepatocyte growth arrest or cell death and were shown to secrete factors that attract and activate hepatic stellate cells for ECM deposition (Richardson *et al*, 2007; Schuppan & Kim, 2013). Moreover, biliary cells were previously suggested to be more resistant to oxidative stress and cell death than hepatocytes (Richardson *et al*, 2007). Thus, their expansion in Casp-8^LPC-KO^ mice might reflect a biliary regenerative response in a context of necroptotic hepatocytes, but might also represent a functional amplification loop in the mediation of RIP3-dependent liver fibrosis upon NASH. Interestingly, our findings indicated that biliary cells seemed to express high levels of RIP3 compared to hepatocytes, a finding currently lacking a functional explanation. It is possible that, given their putative resistance to cell death (Schuppan & Kim, 2013), biliary or precursor cells might tolerate higher levels of RIP3 in the absence of functional Caspase-8 before undergoing cell death. Future experiments with bile duct-specific cre-lines could also reveal a previously unrecognized role of RIP3 or Caspase-8 in biliary homeostasis, regeneration, or the intercellular communication between biliary cells and hepatocytes.

We have recently shown that Caspase-8-dependent apoptosis but not RIP3-dependent necroptosis promoted liver fibrosis and hepatocarcinogenesis in a model of liver injury caused by conditional deletion of the kinase TGF-β-activated Kinase-1 (*Tak1*) in parenchymal liver cells (Vucur *et al*, 2013). In this light, our present findings showing a protective function of Caspase-8 and an injury-promoting role of RIP3 are opposing their roles in the TAK1 model, indicating that activation modes and outcomes of distinct programmed cell death-forms depend on the initiating stimulus and pathogenic context. Given that chemical apoptosis inhibitors have already been tested in clinical studies in patients with NASH (Ratziu *et al*, 2012), pharmacological targeting of the necroptosis pathway might potentially have additive beneficial effects in a combinatory approach together with apoptosis inhibitors that do not specifically target Caspase-8 in these patients. In addition, previous studies showed that serum parameters reflecting activation of apoptosis might serve as biomarkers in NASH (Chakraborty *et al*, 2012). Therefore, enhanced activation of necroptosis could serve as an indicator for more progressive liver disease, which should be evaluated in future prospective studies.

## Materials and Methods

### Study approval

All animal experiments were approved by the Federal Ministry for Nature, Environment and Consumers' Protection of the state of North Rhine-Westphalia and were performed in accordance to the respective national, federal, and institutional regulations.

### Generation of conditional knockout mice

Mice carrying loxP-site-flanked (floxed) alleles of the *Caspase-8*-gene (Caspase-8 fl) (Salmena *et al*, 2003) were crossed to *alfp*-cre transgenic mice (Kellendonk *et al*, 2000) to generate a liver parenchymal cell (LPC)-specific knockout (Caspase-8^LPC-KO^). Mice with constitutive deletion of *RIP3* (RIP3^−/−^) were described before (Newton *et al*, 2004). Mice with double knockout of conditional deletion of *Caspase-8* and constitutive ablation of *Rip3* (Caspase-8^LPC-KO^/RIP3^−/−^) were generated by intercrossing the respective lines. *Alb*-Cre Caspase-8^Floxed^ transgenic mice were described previously (Hatting *et al*, 2013). In all experiments, littermates carrying the respective loxP-flanked alleles but lacking expression of Cre recombinase were used as wild-type (WT) controls. Mice were bred on a C57BL/6 genetic background. Only sex-matched animals were compared.

### Animal experiments

Mice were fed with a methionine choline-deficient (MCD) diet (MP Biomedicals) for 2 weeks (short term) or 8 weeks (long term). Livers from these mice were collected, fixed in 4% PFA, and embedded in paraffin for histological evaluation. Intraperitoneal injection of the JNK-Inhibitor SP600125 (15 μl/1 mg) (Absource Diagnostics) or vehicle (DMSO) was performed twice a day over 2 weeks of MCD feeding.

### Human liver tissue

Human liver biopsy specimens and clinicopathological data were obtained from Newcastle upon Tyne University / Hepatopancreatobiliary and Gastroenterology Research Tissue Bank and Essen University. The project was authorized by the local ethics committees and conducted in accordance with the ethical standards laid down in the Declaration of Helsinki (Newcastle and North Tyneside 1 Research Ethics Committee, Newcastle upon Tyne, Reference number 10/10906/41 and Research Ethics Committee, Essen University, Reference number 09-4252). Histological scoring system for non-alcoholic fatty liver disease (NAFLD) was performed according to the NAS score system (Kleiner *et al*, 2005).

### Serum analysis

Serum ALT, AST, and GLDH activities were measured by standard procedures in the Institute of Clinical Chemistry of the RWTH University Hospital Aachen.

### Triglyceride assay

The intrahepatic triglyceride tenor was measured by TG liquicolor mono (Human Diagnostics) according to the manufacturer's instructions from homogenized frozen liver sample.

### Western blot analysis

Liver tissue was homogenized in NP-40 lysis buffer using a tissue grind pestle (Kimble/Chase) to obtain protein lysates. These were separated by SDS–polyacrylamide gel electrophoresis (PAGE), transferred to PVDF membrane, and analyzed by immunoblotting. Membranes were probed with the following antibodies: anti p-AKT, anti p-ERK, anti p-JNK, anti p-p38 (Cell Signaling), anti-Caspase-8 (Enzo), anti-RIP3 (IMGENEX) and anti-GAPDH (ABD Serotec). As secondary antibodies, anti-rabbit-HRP and anti-mouse-HRP (Amersham) were used.

### Flow cytometry

Multicolor staining was conducted using combinations of the following mAbs: F4/80 (Serotec), CD11b (eBioscience), CD45, and Ly6G (BD). Flow cytometric analysis was performed on a FACS-Canto II (BD). Absolute cell numbers were determined by adding 2 × 104 Calibrite APC beads (BD) to each sample before measurement as internal reference standard. Data were analyzed using FlowJo software (Tree Star). For measurement of MCP-1 in total liver protein extracts, we used the mouse FlowCytomix kit (eBioscience) according to the manufacturer's instructions using a FACSCanto II System (BD).

### Histological examination

Paraffin sections (2 μm) were stained with H/E or various primary and secondary antibodies. Paraformaldehyde (4%)-fixed and paraffin-embedded liver tissue were incubated in Bond Primary antibody diluent (Leica), and staining was performed on a BOND-MAX immunohistochemistry robot (Leica Biosystems) using BOND polymer refine detection solution for DAB. The following antibodies were used: Antibodies against F4/80 (BMA Biomedicals AG, 1:120), anti-CD45 (BD, 1:200) anti-CK19 (TROMAIIIc, Hybridoma bank, 1:500), anti-Ki67 (NeoMarkers; 1:200), anti-cleaved Caspase-3 (Cell Signaling; 1:300), anti-phospho c-Jun (Abcam, 1:100), and anti-RIP3 (Enzo, 1:500). Image acquisition was performed on an Olympus BX53 microscope with a Leica SCN400 slide scanner. The number of hepatocytes positively stained for the different nuclear markers (e.g. Ki67, pc-Jun) was determined using SlidePath TissueIA image analysis software (Leica) on whole tissue sections (paraffin embedded) and normalized to tissue area or hepatocyte number, respectively. Hepatocytes positively stained for cleaved Caspase-3 and RIP3 as well as macrophages positive for F4/80, immune cells positively stained for CD45 or MHCII, and fibrotic fibers stained with Sirius Red were quantified numerically (positive cells per total tissue area) or densitometrically (area stained per total tissue area) using SlidePath TissueIA image analysis software (Leica) on whole tissue sections and normalized to total tissue area.

### Cell culture

L929 cells were cultured in Dulbecco's modified Eagle's medium supplemented with 10% fetal calf serum, penicillin (100 IU/ml), streptomycin (0.1 mg/ml), and l-glutamine (0.03%). Bone marrow cells were isolated from femur and tibia of 8-week-old C57BL6/J and RIP3^−/−^ mice. To obtain fibroblast-conditioned medium (FCM) which is known to contain the macrophage colony-stimulating factor (MCSF, CSF1), L929 fibroblasts were cultured in RPMI medium containing 10% fetal calf serum (FCS) for 3 days, and the supernatant was collected, filtered, and stored until usage at −80°C. For the generation of bone marrow derived macrophages (BMM), bone marrow cells were cultured in RPMI medium containing 10% FCS and 20% FCM for 1 week on bacterial grade plastic plates (Greiner). At day 7, cells were either left untreated or stimulated for additional 24 h with lipopolysaccharides (LPS, 1 μg/ml) (Sigma-Aldrich).

### Analysis of cell survival

L929 cells were cultured overnight in 12-well plates and used at 50% of confluence. After pre-treatment with zVAD (20 μM) (Millipore) for 1 h, the cells were incubated with Necrostatin-1 (10 μM) (Santa Cruz), SP600125 (20 μM) (Absource), or DMSO as control. Nineteen hours later, the cells were incubated with 1 ng/ml of MTT reagent (Life Technologies) for 2 h. Once MTT crystals were developed and controlled under light microscopy, they were dissolved in DMSO and quantified by measuring absorbance at 540 nm.

### Quantitative real-time PCR

Total RNA was purified from liver tissue using TRIzol reagent (Invitrogen) and an RNeasy Mini kit (Qiagen). The quantity and quality of the RNA were determined spectroscopically using a nanodrop (Thermo Scientific). Total RNA (1 μg) was used to synthesize cDNA using the Transcriptor cDNA First-Strand Synthesis Kit (Roche) according to the manufacturer's protocol and was resuspended in 50 μl of H_2_O. cDNA samples (2 μl) were used for real-time PCR in a total volume of 25 μl using SYBR Green Reagent (Invitrogen) and specific primers on a qPCR machine (Applied Biosystems 7300 Sequence Detection System). All real-time PCRs were performed in duplicates. Data were generated and analyzed using SDS 2.3 and RQ manager 1.2 software. Primer sequences are available upon request. All values were normalized to the level of beta-actin mRNA.

### Statistical analysi*s*

Data were analyzed using PRISM software (GraphPad, Inc., La Jolla, CA) and are expressed as SEM. Statistical significance between experimental groups was assessed using unpaired two-sample *t*-test, Mann–Whitney test, and unpaired two-sample *t*-test with Welch's correction (*P < 0.05; **P < 0.01; ***P < 0.001). The exact *P*-values of each experiment and specific tests used are provided in Supplementary Table S1.

**The paper explained**

**Problem**

Apoptosis was previously considered as the main driver of NASH development in patients with fatty liver disease. In this line, Caspase-8 was suggested as a promising target for novel pharmacological strategies, but it was unknown whether other programmed cell-death pathways than apoptosis might be involved in the pathophysiology of NASH.

**Results**

We show that RIP3-dependent necroptosis represents a major driving force mediating NASH-induced liver fibrosis. This function of RIP3 was mediated by activation of JNK as its pharmacological inhibition reduced intrahepatic level of RIP3 and subsequently liver fibrosis and was associated with MCP-1-mediated recruitment of monocytes and an expansion of intrahepatic biliary / progenitor cells. Finally, in contrast to previous assumptions, we demonstrate that the main function of Caspase-8 in this context is to suppress the deleterious effect of RIP3-dependent necroptosis.

**Impact**

The novel RIP3/JNK axis in the liver might represent a novel pharmacological target and/or biomarker in the clinical context of NASH and NASH-induced liver fibrosis. In contrast, pharmacological targeting of Caspase-8 might have deleterious effects in NASH patients, as this might activate necroptosis and trigger liver fibrosis.

## Supporting information

for this article is available online: http://embomolmed.embopress.org
