# Supplementary material for: A positive feedback loop between RIP3 and JNK controls non-alcoholic steatohepatitis
Source: EMBO Mol Med. 2014 Jun 24;6(8):1062–74. doi: 10.15252/emmm.201403856 (PMC4154133; doi:10.15252/emmm.201403856)
Supplement: Supplementary file 1 [file emmm0006-1062-sd1.pdf]

Supporting Information Fig S1

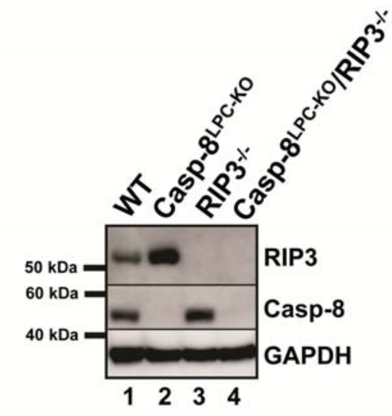

Supp Information Fig S1:

Western blot analysis of whole liver-protein extracts from WT, Caspase-8^{LPC-KO}, Caspase-8^{LPC-KO}/$RIP3^{-/-}$ and $RIP3^{-/-}$ untreated-mice, using antibodies against Caspase-8, RIP3 and GAPDH as loading control.
