# Supplementary material for: A positive feedback loop between RIP3 and JNK controls non-alcoholic steatohepatitis
Source: EMBO Mol Med. 2014 Jun 24;6(8):1062–74. doi: 10.15252/emmm.201403856 (PMC4154133; doi:10.15252/emmm.201403856)
Supplement: Supplementary file 2 [file emmm0006-1062-sd2.pdf]

Supporting Information Fig S2

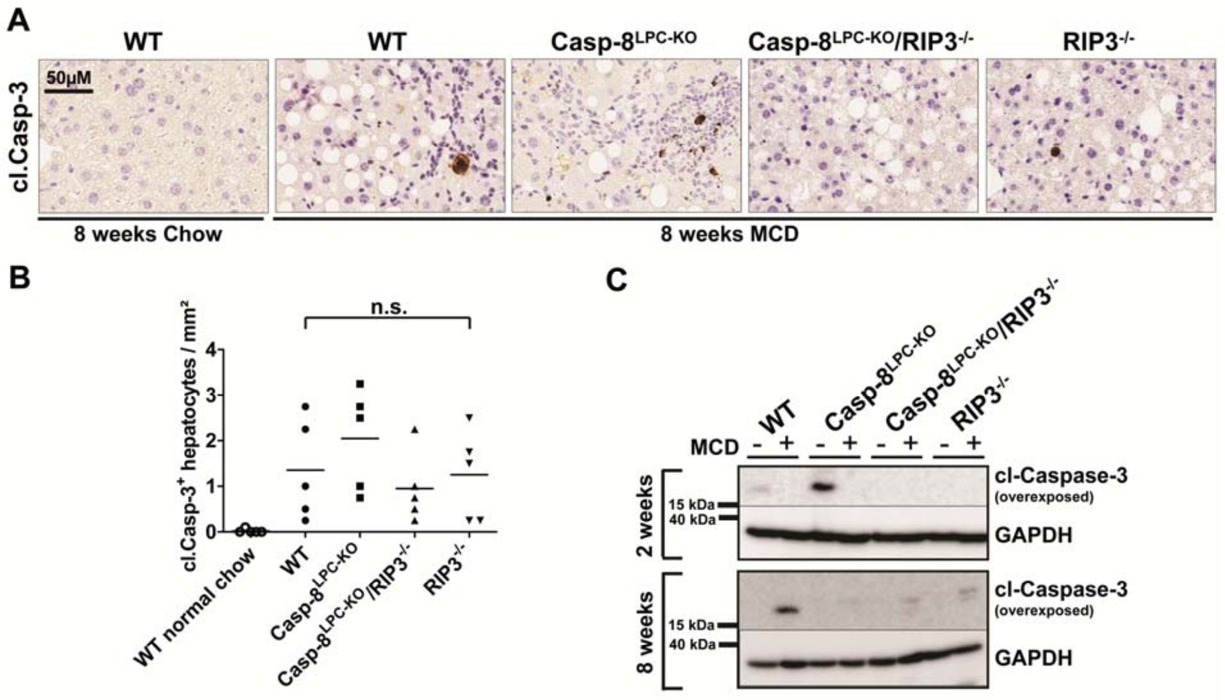

Supporting Information Fig S2: analysis of apoptosis upon MCD-diet feeding.

(**A**) Immunohistochemical analysis of cleaved Caspase-3 on representative liver sections from the indicated mice fed for 8-weeks with MCD-diet or normal chow.

(**B**) Statistical analysis of cleaved Caspase-3^{+} hepatocytes. Results are shown as mean, n=5.

(**C**) Western blot analysis of whole liver-protein extracts from WT, Caspase-8^{LPC-KO}, Caspase-8^{LPC-KO}/RIP3$^{-/-}$, RIP3$^{-/-}$ and untreated-mice, using antibodies against cleaved Caspase-3 and GAPDH as loading control.
