# Supplementary material for: A positive feedback loop between RIP3 and JNK controls non-alcoholic steatohepatitis
Source: EMBO Mol Med. 2014 Jun 24;6(8):1062–74. doi: 10.15252/emmm.201403856 (PMC4154133; doi:10.15252/emmm.201403856)
Supplement: Supplementary file 3 [file emmm0006-1062-sd3.pdf]

Supporting Information Fig S3

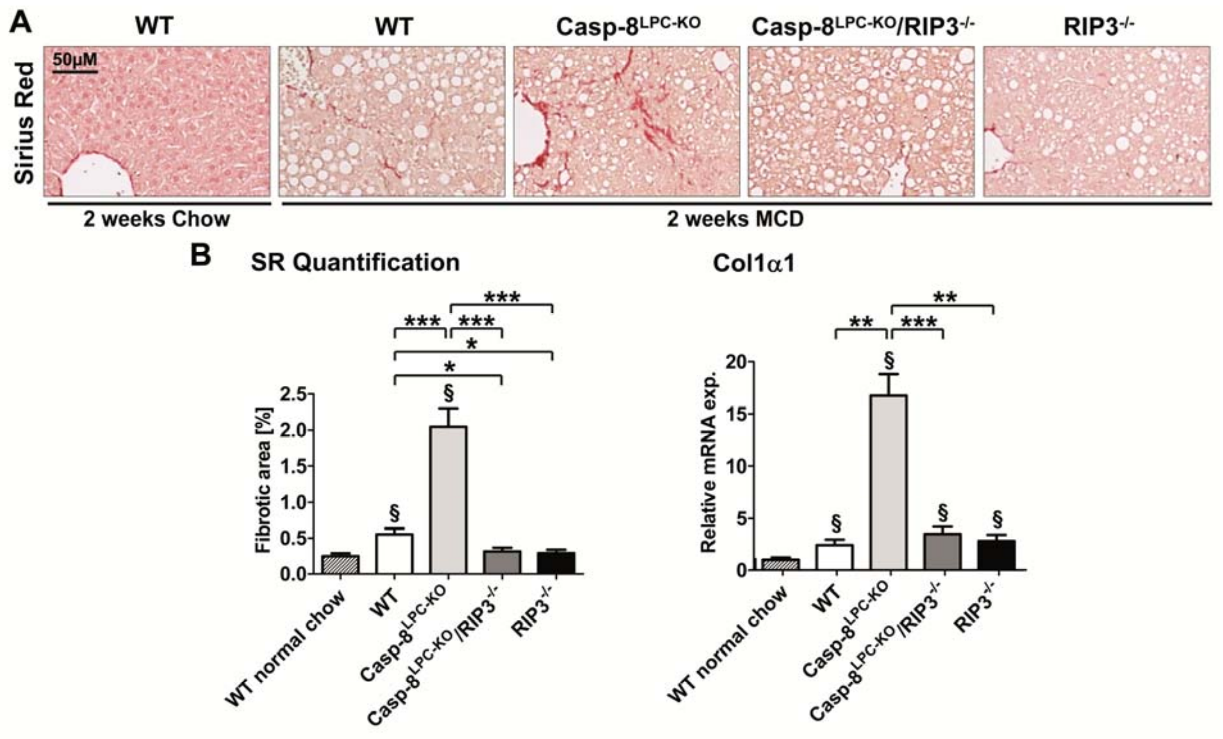

Supporting Information Fig S3: RIP3 mediates the transition from steatosis to MCD-induced fibrosis in Caspase-8-deficient livers.

(**A**) Representative Sirius Red stainings of liver slides from WT, Casp-8^{LPC-KO}, Casp-8^{LPC-KO}/$RIP3^{-/-}$ and $RIP3^{-/-}$ mice fed for two weeks with MCD-diet.

(**B**) Left: statistical quantification of light polarized Sirius Red pictures, results are shown as mean, n=6 per group. Right: *Col1α1* mRNA levels were determined by qRT-PCR. Values were calculated relative to WT mice fed with normal show and β-catenin was used as an internal standard, n=6 per group. § (*P<0.05) indicates that fibrotic area and *Col1α1* are significantly increased from basal WT group.
