# Supplementary material for: A positive feedback loop between RIP3 and JNK controls non-alcoholic steatohepatitis
Source: EMBO Mol Med. 2014 Jun 24;6(8):1062–74. doi: 10.15252/emmm.201403856 (PMC4154133; doi:10.15252/emmm.201403856)
Supplement: Supplementary file 4 [file emmm0006-1062-sd4.pdf]

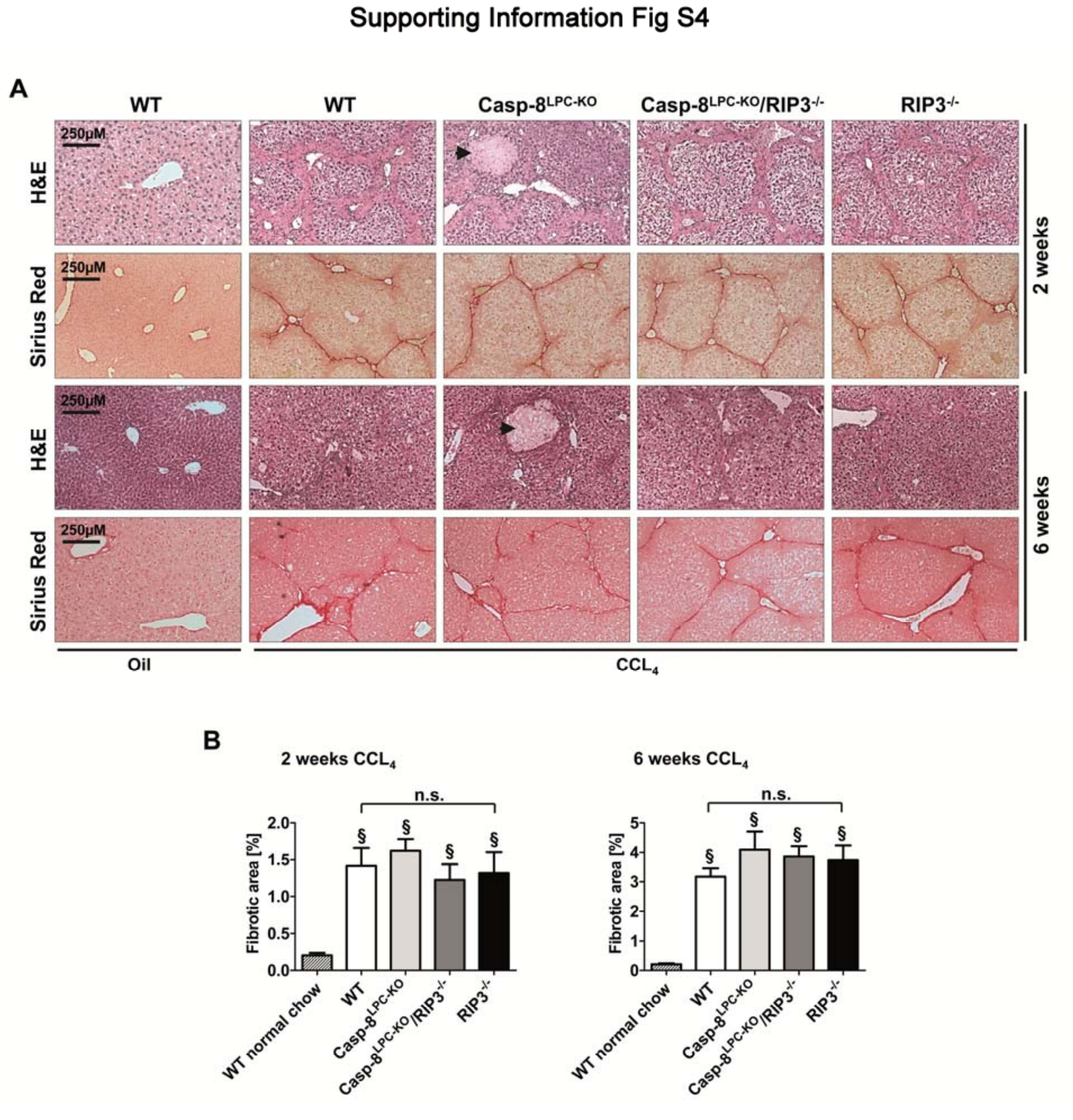

Supporting Information Fig S4: RIP3 deficiency does not protect against liver fibrosis in CCL_4-induced liver injury.

(**A**) Representative H&E and Sirius Red stainings of WT, Casp-8^{LPC-KO}, Casp-8^{LPC-KO}/RIP3$^{-/-}$ and RIP3$^{-/-}$ mice injected twice weekly with carbon tetrachloride (CCL_4) for 2 and 6 weeks. Black arrows indicate necrotic areas seen in Casp-8^{LPC-KO} mice.

(**B**) Statistical quantification of light polarized Sirius Red pictures, results are shown as mean, n=6 per group.
