# Supplementary material for: A positive feedback loop between RIP3 and JNK controls non-alcoholic steatohepatitis
Source: EMBO Mol Med. 2014 Jun 24;6(8):1062–74. doi: 10.15252/emmm.201403856 (PMC4154133; doi:10.15252/emmm.201403856)
Supplement: Supplementary file 5 [file emmm0006-1062-sd5.pdf]

Supporting Information Fig S5

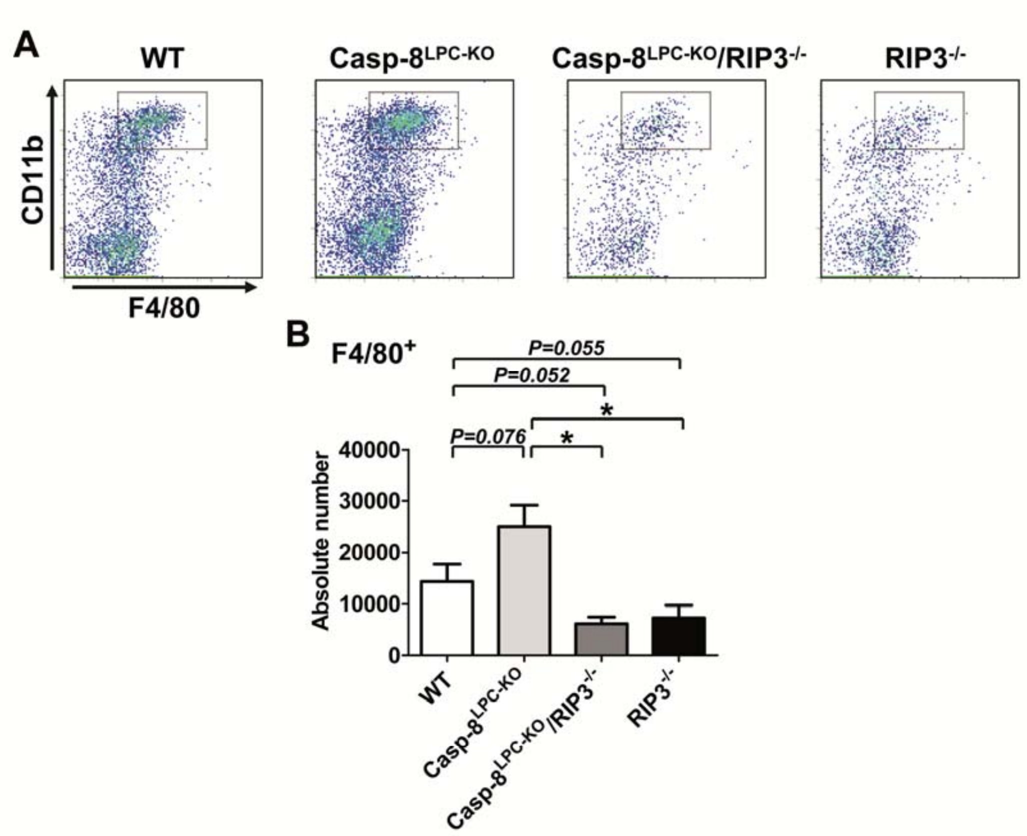


**Supporting Information Fig S5: RIP3 promotes the hepatic recruitment of F4/80$^{+}$ cells.**

(**A**) Representative FACS plots for myelo-monocytic cells (F4/80 $^{+}$) from individual mice of the indicated genotypes after two weeks of MCD-diet feeding.

(**B**) Statistical analysis of FACS data for intrahepatic levels of myelo-monocytic cells in the indicated mouse groups after two weeks of MCD-diet feeding. Results are shown as mean, error bars indicate SEM, n=5.
