# Supplementary material for: A positive feedback loop between RIP3 and JNK controls non-alcoholic steatohepatitis
Source: EMBO Mol Med. 2014 Jun 24;6(8):1062–74. doi: 10.15252/emmm.201403856 (PMC4154133; doi:10.15252/emmm.201403856)
Supplement: Supplementary file 6 [file emmm0006-1062-sd6.pdf]

Supporting Information Fig S6

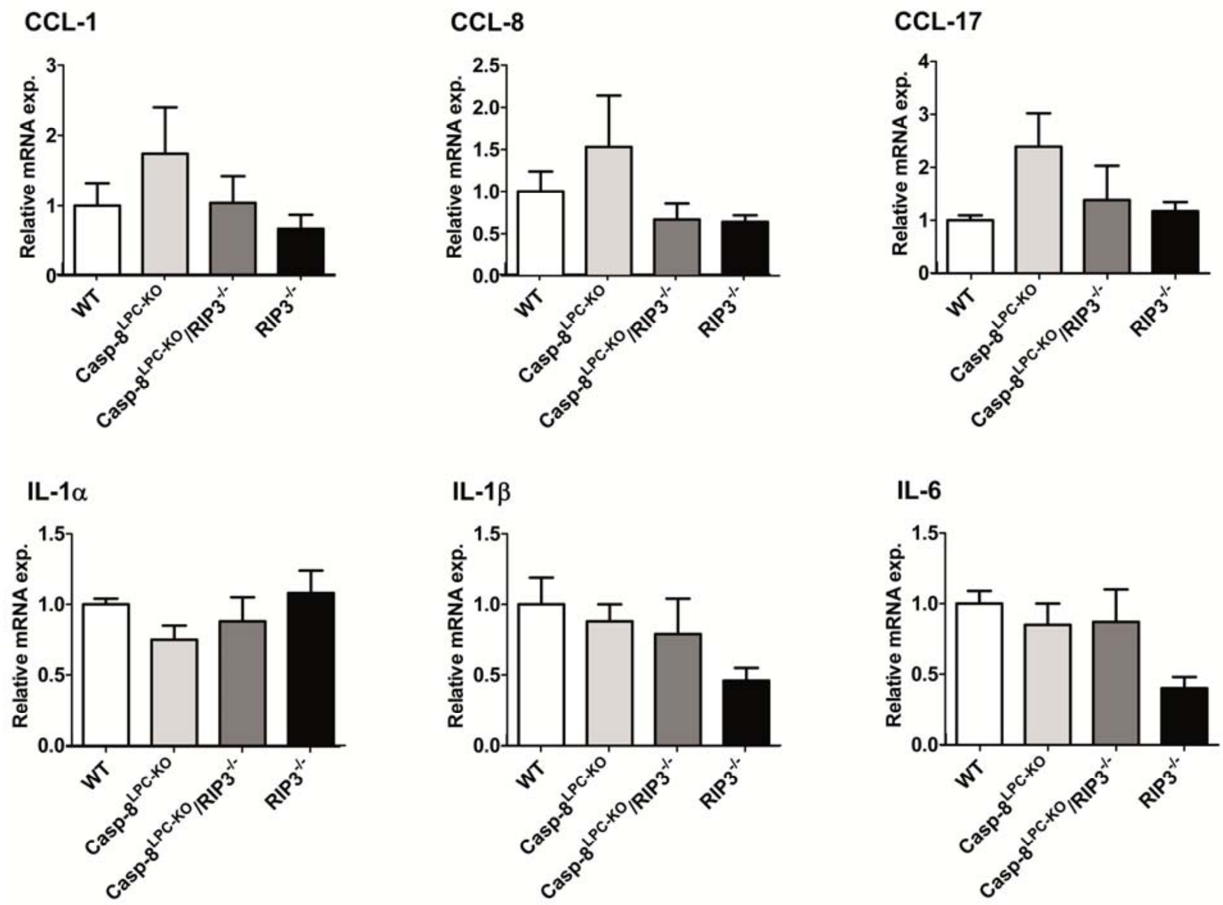

Supporting Information Fig S6: mRNA levels of pro-inflammatory cytokines in mice fed 2 weeks with MCD-diet.

CCL-1, *CCL-8*, *CCL-17*, *IL-1α*, *IL-1β*, *IL-6* mRNA levels were assessed by RT-PCR, n=6 per groups, values were calculated relative to WT mice and β-catenin was used as an internal standard. Error bars represent SEM.
