# Supplementary material for: A positive feedback loop between RIP3 and JNK controls non-alcoholic steatohepatitis
Source: EMBO Mol Med. 2014 Jun 24;6(8):1062–74. doi: 10.15252/emmm.201403856 (PMC4154133; doi:10.15252/emmm.201403856)
Supplement: Supplementary file 7 [file emmm0006-1062-sd7.pdf]

Supporting Information Fig S7

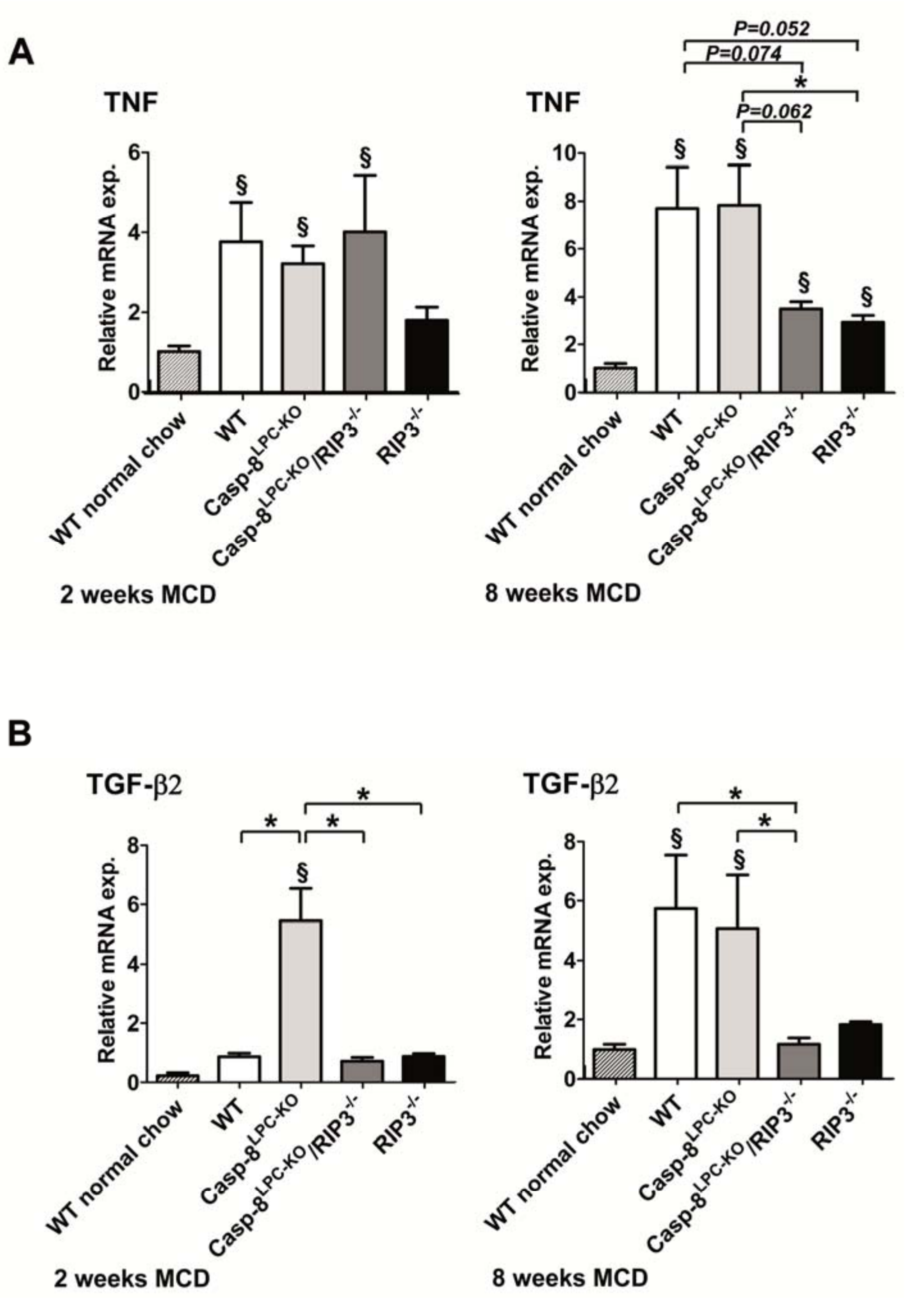

Supporting Information Fig S7: Analysis of TGF-β2 and TNF expression in MCD-induced NASH.

(**A**) *TNF-α* and (**B**) *TGF-β2* mRNA levels were assessed by RT-PCR in the indicated genotypes, values were calculated relative to WT mice fed with normal show and β-catenin was used as an internal standard, n=6 per group. § (*P<0.05) indicates mRNA levels are significantly increased compared to basal values in the WT group.
