# Supplementary material for: A positive feedback loop between RIP3 and JNK controls non-alcoholic steatohepatitis
Source: EMBO Mol Med. 2014 Jun 24;6(8):1062–74. doi: 10.15252/emmm.201403856 (PMC4154133; doi:10.15252/emmm.201403856)
Supplement: Supplementary file 8 [file emmm0006-1062-sd8.pdf]

Supporting Information Fig S8

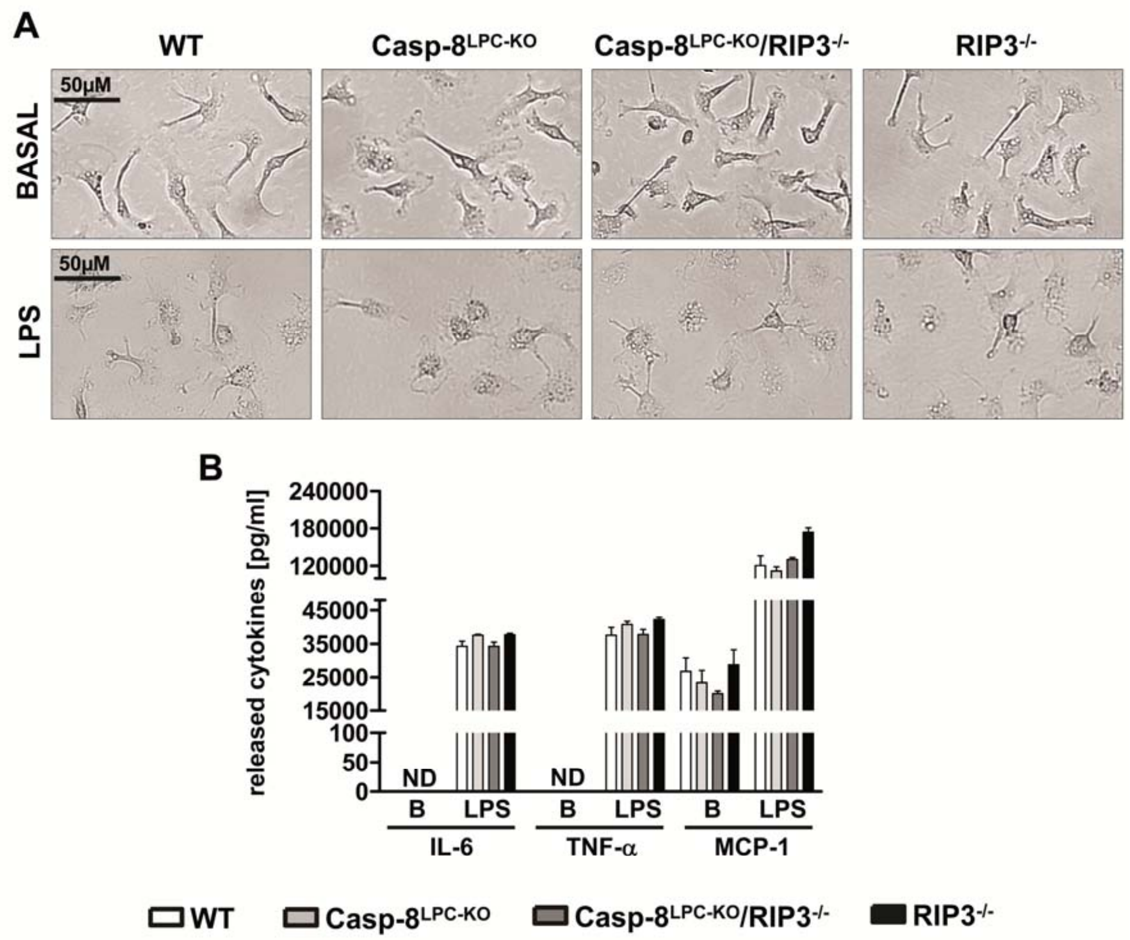

Supporting Information Fig S8: RIP3 deletion in monocytic cells does not lead to impaired immune responses compared to WT mice.

(**A**) Morphological aspects of macrophages of the different groups of mice at basal level and after LPS stimulation.

(**B**) FACS-based micro-beads fluorescence assay for IL-6, TNF-α and MCP-1 expression in liver protein homogenates. Four 10cm plates of WT, Casp-8^{LPC-KO}, Casp-8^{LPC-KO}/RIP3$^{-/-}$ and RIP3$^{-/-}$ macrophages were used for LPS stimulation. Error bars indicate SEM.
