# Supplementary material for: A positive feedback loop between RIP3 and JNK controls non-alcoholic steatohepatitis
Source: EMBO Mol Med. 2014 Jun 24;6(8):1062–74. doi: 10.15252/emmm.201403856 (PMC4154133; doi:10.15252/emmm.201403856)
Supplement: Supplementary file 9 [file emmm0006-1062-sd9.pdf]

Supporting Information Fig S9

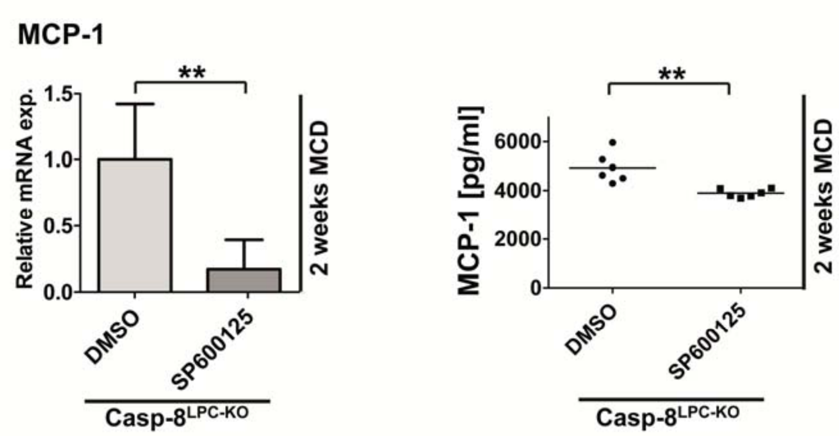

Supporting Information Fig S9: RIP3 controls MCP-1 release through activation of Jun-(N)-terminal kinase (JNK).

Left: *MCP-1* mRNA levels were assessed by RT-PCR, n= 6 per group, values were calculated relative to Casp-8$^{LPC\text{-}KO}$ mice treated with DMSO and β-catenin was used as an internal standard, n=6 per group. Right: FACS-based micro-beads fluorescence assay for MCP-1 expression in liver protein homogenates, n=6 per group.
