# Supplementary material for: A positive feedback loop between RIP3 and JNK controls non-alcoholic steatohepatitis
Source: EMBO Mol Med. 2014 Jun 24;6(8):1062–74. doi: 10.15252/emmm.201403856 (PMC4154133; doi:10.15252/emmm.201403856)
Supplement: Supplementary file 10 [file emmm0006-1062-sd10.pdf]

Supporting Information Fig S10

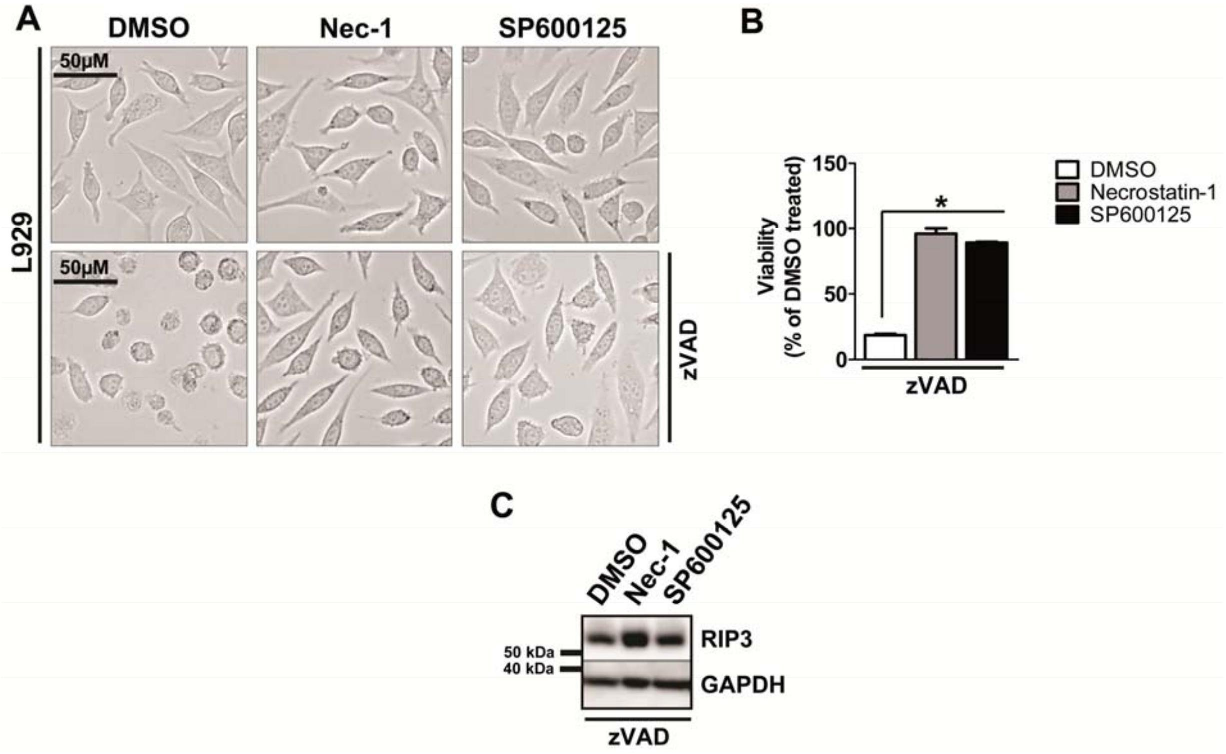

Supporting Information Fig S10: JNK inhibition blocks zVAD-induced necroptosis and reduces expression levels of RIP3.

(**A**) Morphological features of L929 cells treated for 19h with zVAD, Nec-1 and SP600125 as indicated.

(**B**) Cell survival analysis using a MTT assay after 19 hours of zVAD stimulation of L929 cells additionally treated with Nec-1, SP600125 or DMSO as a control. Error bars indicate SEM of duplicates and data are representative of at least three independent experiments.

(**C**) Western blot analysis of L929 cells treated with the indicated substances for 8 hours, using antibodies against RIP3 and GAPDH as loading control.
