# Supplementary material for: A positive feedback loop between RIP3 and JNK controls non-alcoholic steatohepatitis
Source: EMBO Mol Med. 2014 Jun 24;6(8):1062–74. doi: 10.15252/emmm.201403856 (PMC4154133; doi:10.15252/emmm.201403856)
Supplement: Supplementary file 11 [file emmm0006-1062-sd11.pdf]

Supporting Information Fig S11

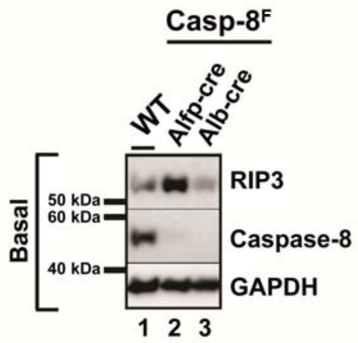

Supporting Information Fig S11: RIP3 is over-expressed in Alfp-cre mice compared to Alb-cre mice.

Western blot analysis of whole liver-protein extracts from WT, Alfp-cre- and Alb-cre-Caspase-8^{Floxed} mice, using antibodies against Caspase-8, RIP3 and GAPDH as loading control.
