# Supplementary material for: A positive feedback loop between RIP3 and JNK controls non-alcoholic steatohepatitis
Source: EMBO Mol Med. 2014 Jun 24;6(8):1062–74. doi: 10.15252/emmm.201403856 (PMC4154133; doi:10.15252/emmm.201403856)
Supplement: Supplementary file 12 [file emmm0006-1062-sd12.pdf]

Supplementary Table S1

Listing of exact *p* -Values and Statistical Tests in each sub-Figure

**Figure 1A**	**AST**	***p* values**	
2 weeks Chow	C8-WT	**0,0292**	Mann Whitney test
	C8-C8R3	**0,009**	Unpaired t-test
	C8-R3	**0,0295**	Unpaired t-test
2 weeks MCD	C8-WT	**0,0119**	Mann Whitney test
	C8-C8R3	**0,0015**	Unpaired t test with Welch's correction
	C8-R3	**0,0019**	Unpaired t test with Welch's correction
	WT-C8R3	**0,05**	Mann Whitney test
	WT-R3	**0,0422**	Mann Whitney test
8 weeks MCD	C8-WT	**0,0079**	Mann Whitney test
	C8-C8R3	**0,0119**	Mann Whitney test
	C8-R3	**0,0119**	Mann Whitney test
	WT-C8R3	**0,0062**	Unpaired t-test
	WT-R3	**0,0098**	Unpaired t test with Welch's correction
2w MCD vs 2w Chow	WT-WT	**0,0004**	Mann Whitney test
	C8-C8	**0,0001**	Unpaired t test with Welch's correction
	C8R3-C8R3	**0,0001**	Unpaired t test with Welch's correction
	R3-R3	**0,0001**	Unpaired t test with Welch's correction
	**ALT**	***p* values**	
2 weeks MCD	C8-WT	**0,0312**	Unpaired t test with Welch's correction
	C8-C8R3	**0,0091**	Unpaired t test with Welch's correction
	C8-R3	**0,0071**	Unpaired t test with Welch's correction
8 weeks MCD	C8-WT	**0,0128**	Unpaired t-test
	C8-C8R3	**0,0001**	Unpaired t-test
	C8-R3	**0,0001**	Unpaired t-test
	WT-C8R3	**0,0377**	Unpaired t-test
	WT-R3	**0,0422**	Unpaired t test with Welch's correction
2w MCD vs 2w Chow	WT-WT	**0,0001**	Unpaired t test with Welch's correction
	C8-C8	**0,0002**	Unpaired t test with Welch's correction
	C8R3-C8R3	**0,0022**	Mann Whitney test
	R3-R3	**0,0005**	Mann Whitney test
	**GLDH**	***p* values**	
2 weeks Chow	C8-WT	**0,0149**	Unpaired t test with Welch's correction
	C8-C8R3	**0,0154**	Unpaired t test with Welch's correction
	C8-R3	**0,0159**	Unpaired t test with Welch's correction
2 weeks MCD	C8-WT	**0,0039**	Mann Whitney test

	C8-C8R3	**0,0048**	Mann Whitney test
	C8-R3	**0,0006**	Mann Whitney test
8 weeks MCD	C8-WT	**0,0017**	Unpaired t-test
	C8-C8R3	**0,0001**	Unpaired t-test
	C8-R3	**0,0002**	Unpaired t test with Welch's correction
	WT-C8R3	**0,0026**	Unpaired t-test
	WT-R3	**0,0071**	Unpaired t test with Welch's correction
2w MCD vs 2w Chow	WT-WT	**0,0004**	Mann Whitney test
	C8-C8	**0,0057**	Mann Whitney test
	C8R3-C8R3	**0,0001**	Unpaired t test with Welch's correction
	R3-R3	**0,0005**	Mann Whitney test
**Figure 1D**	**RIP3**	***p* values**	
	C8-WT	**0,0115**	Unpaired t test with Welch's correction
	C8-C8R3	**0,0069**	Unpaired t test with Welch's correction
	C8-R3	**0,0068**	Unpaired t test with Welch's correction
	WT-C8R3	**0,0008**	Unpaired t-test
	WT-R3	**0,0042**	Unpaired t test with Welch's correction
	WT-WTnc	**0,0179**	Mann Whitney test
	C8-WTnc	**0,0179**	Mann Whitney test
	**Ki67**	***p* values**	
	C8-C8R3	**0,0089**	Unpaired t test with Welch's correction
	C8-R3	**0,0016**	Unpaired t-test
	WT-C8R3	**0,0003**	Unpaired t-test
	WT-R3	**0,0005**	Unpaired t-test
	WT-WTnc	**0,0026**	Unpaired t-test
	C8-WTnc	**0,0043**	Unpaired t-test
**Figure 2B**	**TG**	***p* values**	
8 weeks MCD	WT-C8R3	**0,0365**	Unpaired t-test
**Figure 2D**	**Fibrotic Area**	***p* values**	
	C8-WT	**0,0366**	Unpaired t-test
	C8-C8R3	**0,0057**	Unpaired t test with Welch's correction
	C8-R3	**0,0062**	Unpaired t test with Welch's correction
	WT-C8R3	**0,0417**	Unpaired t test with Welch's correction
	WT-R3	**0,0483**	Unpaired t test with Welch's correction
	WT-WTnc	**0,0402**	Unpaired t test with Welch's correction
	C8-WTnc	**0,0056**	Unpaired t test with Welch's correction
	Col1 α1	***p* values**	
	C8-WT	**0,0307**	Unpaired t test with Welch's correction
	C8-C8R3	**0,0478**	Unpaired t test with Welch's correction

	C8-R3	**0,045**	Unpaired t test with Welch's correction
	WT-C8R3	**0,0313**	Unpaired t test with Welch's correction
	WT-R3	**0,0426**	Unpaired t test with Welch's correction
	WT-WTnc	**0,0373**	Unpaired t test with Welch's correction
	C8-WTnc	**0,0438**	Unpaired t test with Welch's correction
**Figure 3B**	**CD45**	***p* values**	
	C8-WT	**0,0408**	Unpaired t test with Welch's correction
	C8-C8R3	**0,0252**	Unpaired t test with Welch's correction
	**F4/80**	***p* values**	
	C8-C8R3	**0,0302**	Unpaired t test with Welch's correction
	C8-R3	**0,0355**	Unpaired t test with Welch's correction
	WT-C8R3	**0,0001**	Unpaired t-test
	WT-R3	**0,0001**	Unpaired t-test
	WT-WTnc	**0,0097**	Mann Whitney test
	C8-WTnc	**0,0097**	Mann Whitney test
**Figure 3C**	***MCP-1***	***p* values**	
	C8-WT	**0,0079**	Unpaired t-test
	C8-C8R3	**0,0119**	Unpaired t-test
	C8-R3	**0,008**	Unpaired t-test
	WT-R3	**0,0173**	Unpaired t-test
	C8R3-R3	**0,0173**	Unpaired t-test
	WT-WTnc	**0,0237**	Unpaired t test with Welch's correction
	C8-WTnc	**0,0001**	Unpaired t test with Welch's correction
	**MCP-1**	***p* values**	
	C8-WT	**0,0215**	Unpaired t test with Welch's correction
	C8-C8R3	**0,0098**	Unpaired t test with Welch's correction
	C8-R3	**0,006**	Unpaired t test with Welch's correction
	WT-C8R3	**0,0388**	Unpaired t-test
	WT-R3	**0,036**	Unpaired t-test
	WT-WTnc	**0,0039**	Unpaired t test with Welch's correction
	C8-WTnc	**0,0045**	Unpaired t test with Welch's correction
	C8R3-WTnc	**0,0052**	Unpaired t test with Welch's correction
**Figure 3D**	***MCP-1***	***p* values**	
	C8-C8R3	**0,0295**	Unpaired t test with Welch's correction
	C8-R3	**0,034**	Unpaired t test with Welch's correction
	WT-C8R3	**0,0226**	Unpaired t test with Welch's correction
	WT-R3	**0,0243**	Unpaired t test with Welch's correction
	WT-WTnc	**0,0018**	Unpaired t test with Welch's correction
	C8-WTnc	**0,0121**	Unpaired t test with Welch's correction
	C8R3-WTnc	**0,0483**	Unpaired t test with Welch's correction
	R3-WTnc	**0,0029**	Unpaired t test with Welch's correction

	**MCP-1**	***p* values**	
	C8-WT	**0,0465**	Mann Whitney test
	C8-C8R3	**0,0159**	Mann Whitney test
	C8-R3	**0,0079**	Mann Whitney test
	WT-R3	**0,0003**	Unpaired t-test
	WT-WTnc	**0,0039**	Unpaired t test with Welch's correction
	C8-WTnc	**0,0022**	Mann Whitney test
**Figure 4B**	**CK-19**	***p* values**	
	C8-WT	**0,0259**	Unpaired t-test
	C8-C8R3	**0,025**	Unpaired t-test
	C8-R3	**0,0084**	Unpaired t-test
	C8-WTnc	**0,0237**	Unpaired t test with Welch's correction
**Figure 6B**	**p-cJUN**	***p* values**	
	DMSO-SP6	**0,0056**	Unpaired t test with Welch's correction
**Figure 6C**	AST	***p* values**	
	DMSO-SP6	**0,0135**	Unpaired t test with Welch's correction
	ALT	***p* values**	
	DMSO-SP6	**0,0437**	Unpaired t test with Welch's correction
	GLDH	***p* values**	
	DMSO-SP6	**0,0127**	Unpaired t test with Welch's correction
**Figure 6D**	***Col1* α*1***	***p* values**	
	DMSO-SP6	**0,021**	Unpaired t test
**Figure 6E**	**CD45**	***p* values**	
	DMSO-SP6	**0,043**	Unpaired t test with Welch's correction
	**F4/80**	***p* values**	
	DMSO-SP6	**0,0103**	Unpaired t-test
**Sup Fig 3B**	**Fibrotic Area**	***p* values**	
	C8-WT	**0,0001**	Mann Whitney test
	C8-C8R3	**0,0001**	Mann Whitney test
	C8-R3	**0,0001**	Mann Whitney test
	WT-C8R3	**0,0191**	Unpaired t test
	WT-R3	**0,0112**	Unpaired t test with Welch's correction
	WT-WTnc	**0,003**	Unpaired t test with Welch's correction
	C8-WTnc	**0,0001**	Mann Whitney test
	Col1* α*1	***p* values**	
	C8-WT	**0,0026**	Unpaired t test with Welch's correction
	C8-C8R3	**0,0001**	Unpaired t-test
	C8-R3	**0,003**	Unpaired t test with Welch's correction

	WT-WTnc	**0,0306**	Unpaired t-test
	C8-WTnc	**0,0016**	Unpaired t test with Welch's correction
	C8R3-WTnc	**0,0206**	Unpaired t test with Welch's correction
	R3-WTnc	**0,0283**	Unpaired t test with Welch's correction
**Sup Fig 4B**	**CCL4 2w**	***p* values**	
	WT-WTnc	**0,0007**	Unpaired t test with Welch's correction
	C8-WTnc	**0,0025**	Mann Whitney test
	C8R3-WTnc	**0,0031**	Unpaired t test with Welch's correction
	R3-WTnc	**0,0188**	Unpaired t test with Welch's correction
	**CCL4 8w**	***p* values**	
	WT-WTnc	**0,0001**	Unpaired t test with Welch's correction
	C8-WTnc	**0,0014**	Unpaired t test with Welch's correction
	C8R3-WTnc	**0,0073**	Unpaired t test with Welch's correction
	R3-WTnc	**0,0001**	Unpaired t test with Welch's correction
**Sup Fig 5B**	**F4/80**	***p* values**	
	C8-C8R3	**0,0116**	Unpaired t test with Welch's correction
	C8-R3	**0,0159**	Mann Whitney test
**Sup Fig 7A**	***TNF* 2w**	***p* values**	
	WT-WTnc	**0,0104**	Mann Whitney test
	C8-WTnc	**0,0043**	Mann Whitney test
	C8R3-WTnc	**0,0043**	Mann Whitney test
	***TNF* 8w**	***p* values**	
	WT-WTnc	**0,0181**	Unpaired t test with Welch's correction
	C8-WTnc	**0,0151**	Unpaired t test with Welch's correction
	C8R3-WTnc	**0,0001**	Unpaired t test
	R3-WTnc	**0,0005**	Unpaired t test
	C8-R3	**0,0261**	Unpaired t test
**Sup Fig 7B**	***TGF-β2* 2w**	***p* values**	
	C8-WT	**0,0139**	Unpaired t test with Welch's correction
	C8-C8R3	**0,0124**	Unpaired t test with Welch's correction
	C8-R3	**0,0138**	Unpaired t test with Welch's correction
	C8-WTnc	**0,0151**	Unpaired t test with Welch's correction
	***TGF-β2* 8w**	***p* values**	
	C8-C8R3	**0,0159**	Mann Whitney test
	WT-C8R3	**0,0079**	Mann Whitney test
	WT-WTnc	**0,0043**	Mann Whitney test
	C8-WTnc	**0,0043**	Mann Whitney test
**Sup Fig 9**	***MCP-1***	***p* values**	

DMSO-SP6	**0,0055**	Unpaired t test with Welch's correction

**MCP-1**	***p* values**	
DMSO-SP6	**0,01**	Unpaired t test with Welch's correction
