# Supplementary figures and images for: A positive feedback loop between RIP3 and JNK controls non-alcoholic steatohepatitis
Source: EMBO Mol Med. 2014 Jun 24;6(8):1062–74. doi: 10.15252/emmm.201403856 (PMC4154133; doi:10.15252/emmm.201403856)

RIP3

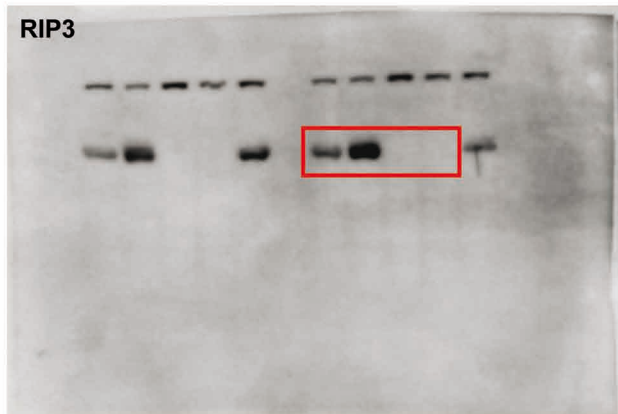

GAPDH

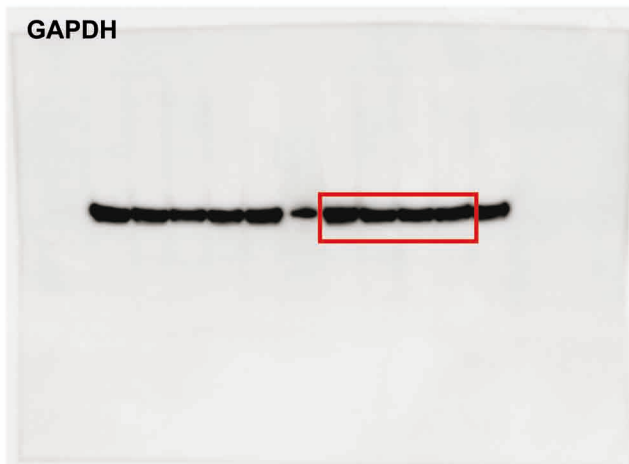

Casp-8

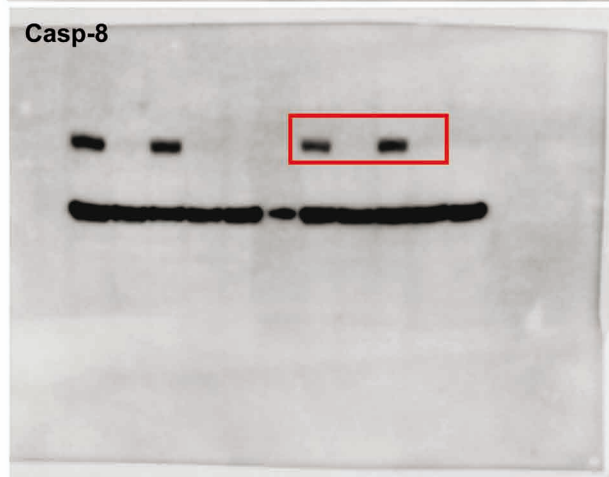

Supplement: Supplementary file 13 [file emmm0006-1062-sd13.pdf]

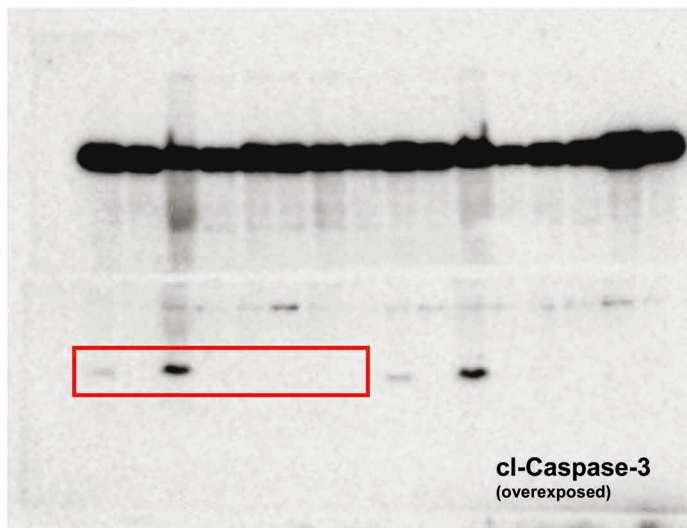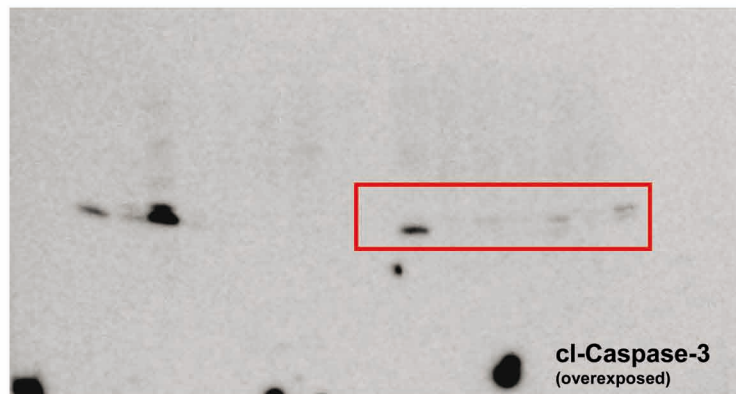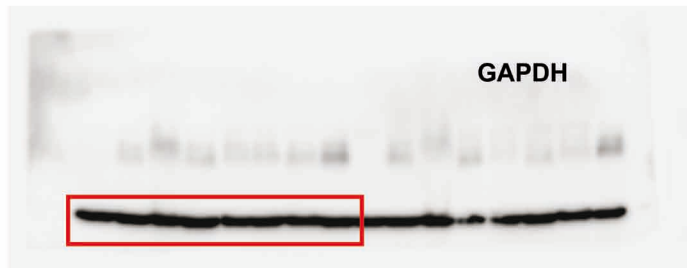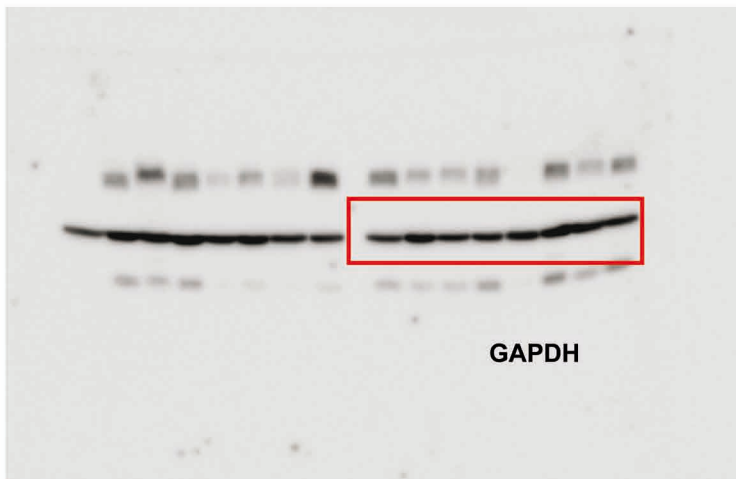

Supplement: Supplementary file 14 [file emmm0006-1062-sd14.pdf]

**RIP3**

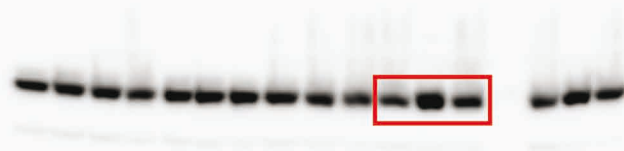

**GAPDH**

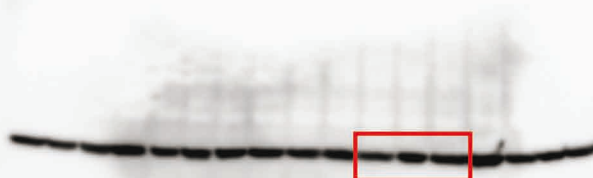

Supplement: Supplementary file 15 [file emmm0006-1062-sd15.pdf]

**RIP3**

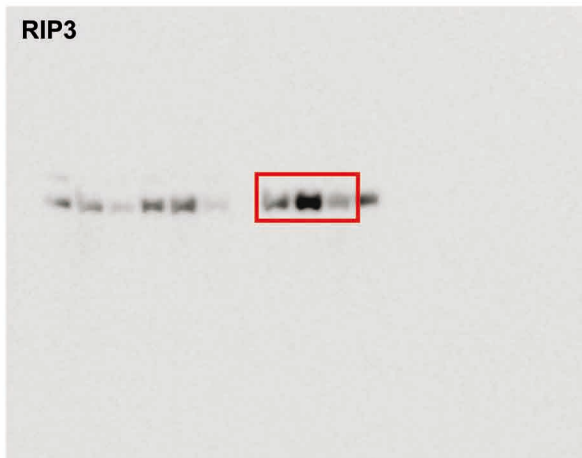

**GAPDH**

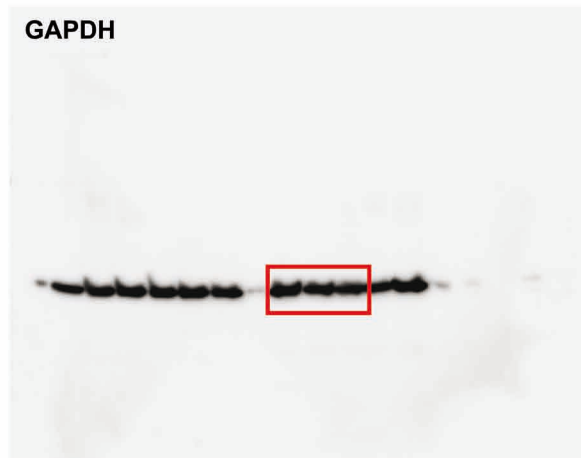

**Caspase-8**

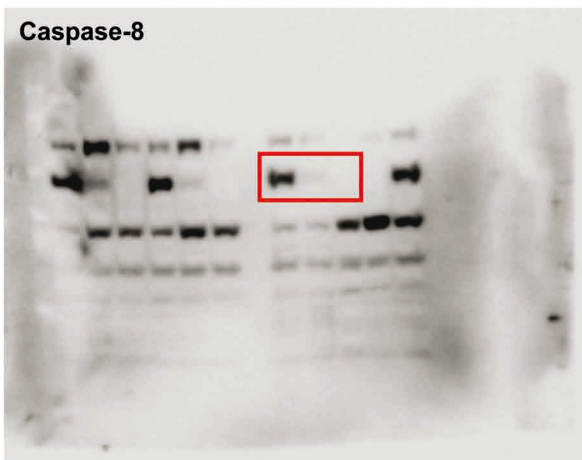

Supplement: Supplementary file 16 [file emmm0006-1062-sd16.pdf]

RIP3

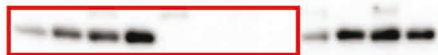

GAPDH

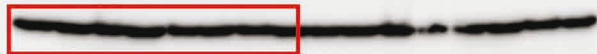

Caspase-8

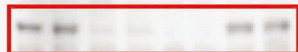

RIP3

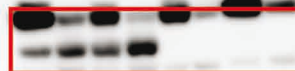

Caspase-8

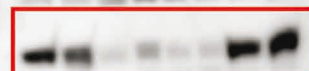

GAPDH

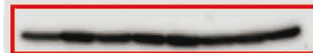

Figure 1

Supplement: Supplementary file 18 [file emmm0006-1062-sd18.pdf]

**RIP3**

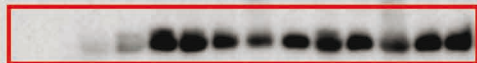

**cl-Caspase-3**  
(overexposed)

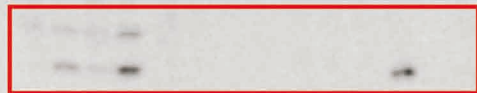

**GAPDH**

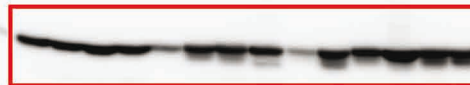

**Figure 5**

Supplement: Supplementary file 19 [file emmm0006-1062-sd19.pdf]

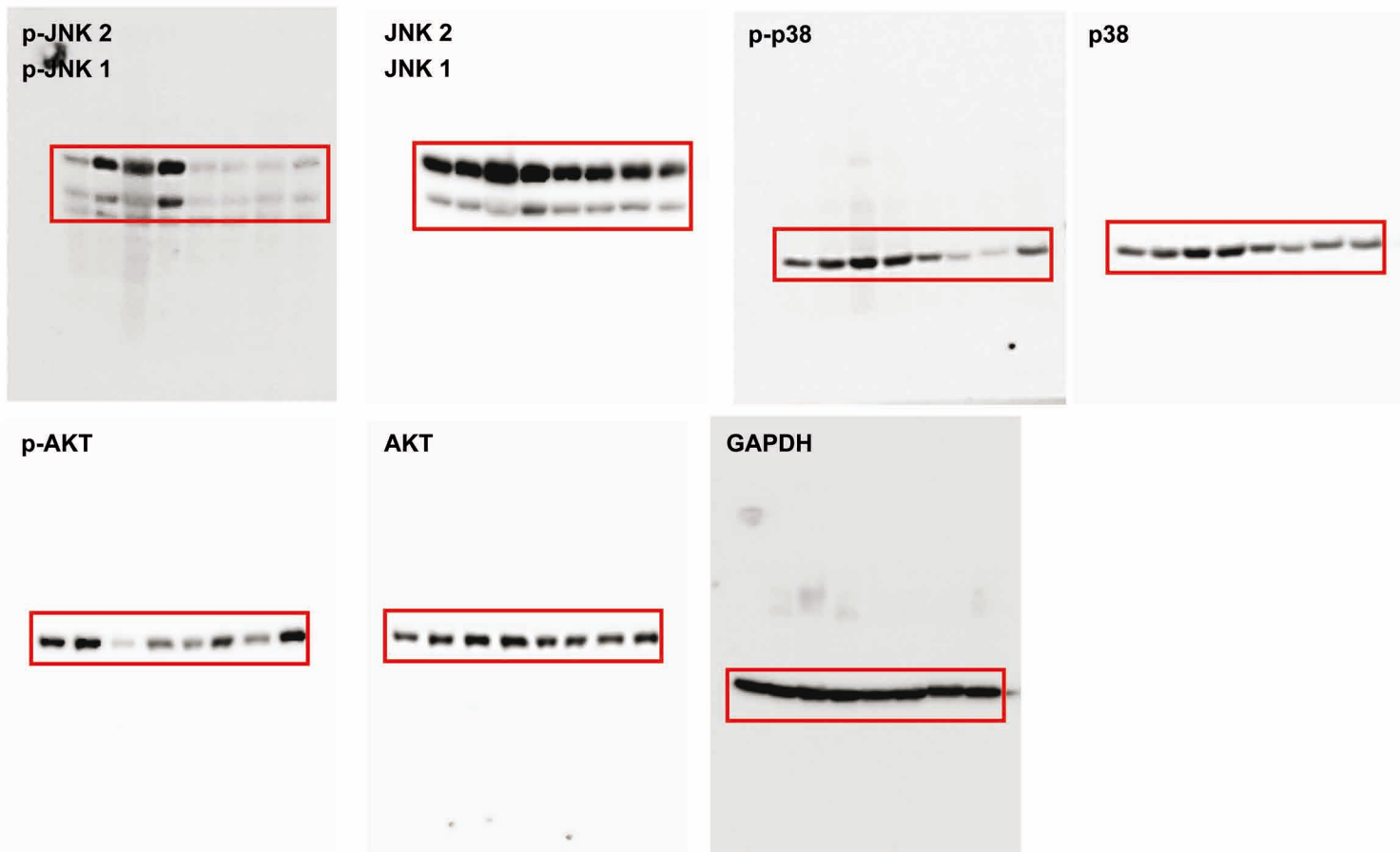

Figure 6A

RIP3

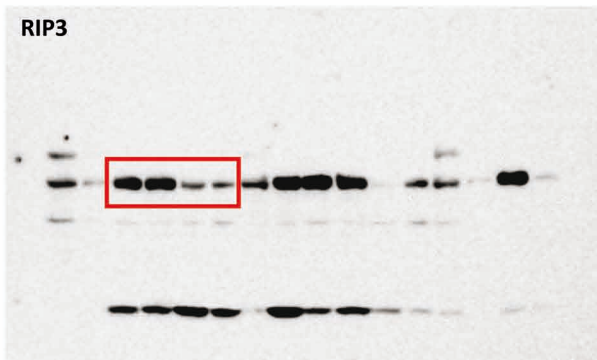

GAPDH

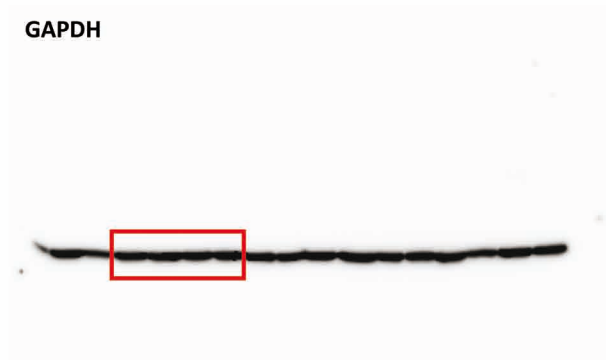

Figure 6F

Supplement: Supplementary file 20 [file emmm0006-1062-sd20.pdf]
