# Supplementary material for: A positive feedback loop between RIP3 and JNK controls non-alcoholic steatohepatitis
Source: EMBO Mol Med. 2014 Jun 24;6(8):1062–74. doi: 10.15252/emmm.201403856 (PMC4154133; doi:10.15252/emmm.201403856)
Supplement: Supplementary file 17 [file emmm0006-1062-sd17.pdf]

# A positive feedback-loop between RIP3 and JNK controls Non-Alcoholic-Steatohepatitis

Jérémie Gautheron, Mihael Vucur, Florian Reisinger, David Vargas Cardenas, Christoph Roderburg, Christiane Koppe, Karina Kreggenwinkel, Anne Theres Schneider, Matthias Bartneck, Ulf Peter Neumann, Ali Canbay, Helen Louise Reeves, Mark Luedde, Frank Tacke, Christian Trautwein, Mathias Heikenwalder and Tom Luedde

*Corresponding author: Tom Luedde, University Hospital Aachen*

**Review timeline:**

| | |
|---|---|
| Submission date: | 15 January 2014 |
| Editorial Decision: | 10 February 2014 |
| Revision received: | 15 April 2014 |
| Editorial Decision: | 19 May 2014 |
| Revision received: | 22 May 2014 |
| Accepted: | 22 May 2014 |

## Transaction Report:

(Note: With the exception of the correction of typographical or spelling errors that could be a source of ambiguity, letters and reports are not edited. The original formatting of letters and referee reports may not be reflected in this compilation.)

*Editor: Roberto Buccione*

1st Editorial Decision 10 February 2014

Thank you for the submission of your manuscript to EMBO Molecular Medicine. We have now heard back from the three Reviewers whom we asked to evaluate your manuscript.

You will see that while two Reviewers are more supportive of your work (with varying degrees of criticism), one is quite negative. All considered, significant issues are raised that prevent us from considering publication at this time. I will not dwell into much detail, as the evaluations are detailed and self-explanatory and will just mention a few main points.

Reviewer 1 is clearly more supportive of your work, but does point out a number of flaws in interpretation and insufficient experimental support for some conclusions. I would like to point out that this Reviewer specifically mentions the need for NASH-specific clinical data to support your conclusions. I must say that I fully agree here, especially given the criticisms of Reviewer 3 (see later below) and the focus of EMBO Molecular Medicine.

Reviewer 2 is especially concerned about the lack of mechanistic support for some important aspects. Reviewer 2 provides more systematic and detailed analysis in this respect, but essentially is in agreement with Reviewer 1. Although I will not be requiring you to perform all the suggested experiments (provided the issues raised are carefully dealt with), I would, however encourage you to develop your study as far as realistically possible in a mechanistic sense for your next, revised version to strengthen your findings and increase their impact. For instance, TUNEL staining might

not be a vital issue in this case.

Reviewer 3 directly questions the validity of the mouse model used and many of his/her criticisms stem from this major point. I must admit that s/he does raise some compelling points, which I would ask you to address directly. As mentioned above, if you could provide clinical data supporting the pathways you outline in this manuscript, the model issue would be superseded. This Reviewer also lists other very important experimental shortcomings and requests for clarification (including discrepancies with respect to previous work) that require your action.

Considered all the above, while publication of the paper cannot be considered at this stage, we would be prepared to consider a substantially revised submission, with the understanding that the Reviewers' concerns must be fully addressed with additional experimental data where appropriate and that acceptance of the manuscript will entail a second round of review.

Since the required revision in this case appears to require a significant amount of time, additional work and experimentation and might be technically challenging, I would understand if you chose to rather seek publication elsewhere at this stage. Should you do so, we would welcome a message to this effect.

Please note that it is EMBO Molecular Medicine policy to allow a single round of revision only and that, therefore, acceptance or rejection of the manuscript will depend on the completeness of your responses included in the next, final version of the manuscript.

As you know, EMBO Molecular Medicine has a "scooping protection" policy, whereby similar findings that are published by others during review or revision are not a criterion for rejection. However, I do ask you to get in touch with us after three months if you have not completed your revision, to update us on the status. Please also contact us as soon as possible if similar work is published elsewhere.

***** Reviewer's comments *****

Referee #1 (Comments on Novelty/Model System):

Best test organism available at the current time.

Referee #1 (Remarks):

The studies of Guatheron & Vucur, et al., detailed here describe the impact of genetic RIP3 or caspase-8 loss on the acquisition of steatotic disease in mouse liver. The authors link RIPK3 and JNK along a pathway that promotes disease, and show that genetic or pharmacologic inhibition (respectively) can serve to ameliorate the symptoms of disease. The studies are well-performed, and document a new role for necroptosis as a driving mechanism in their model of nonalcoholic steatosis. However, there are several points that the authors will want to address, to provide a clear interpretation for their readers as well as for those focused on this field.

In figure 3, MCP1 expression is not impacted by loss of C8 expression - though one would expect the difference seen at 2w to continue. Loss of caspase-8 does increase CD45 recruitment and has been linked to inflammation in other tissues, such as skin. Is MCP1 really implicated in the process? Did the authors look at a 'gold standard' cytokine as a comparator, such as TNFa? This is particularly important, as there are reports that TNFa secretion is critically dependent upon RIPK3 - and central to the induction of necroptosis. Its effects on the monocyte compartment are well known.

The authors refer to RIP3 or Casp-8 activation, but they never provide any evidence that catalytic activity of either enzyme is required for the phenotypes show. In fact, it is linked only to relative expression levels in these studies, and therefore a reader would not be able to link catalytic activity as 'required' in the models or not. In the absence of additional evidence, the text should be amended to indicate expression, rather than activity.

The lack of effect in the immune compartment of the cells at the mRNA level is interesting - the lack of arginase production (and TNF ) after IFN treatement of the RIP3 -/- appears to be significant. However, since these studies were done in RIP3 -/- mice, and not the (more correct) Casp8 LPC/ RIP3-/- (which may be different), the results remain supportive but not conclusive. Does a survey of proteins (via whatever means) grossly suggest concordance with mRNA levels?

In the final figure, the authors look at phospho-protein expression, but of course this data is not interpretable without control blots showing the total of each protein loaded (ie., JNK, p38, etc...).

It should be noted that the effect of systemic administration of a JNK inhibitor may well be on the bone marrow rather than the liver, so it is not clear that the pathway is critically involved in the liver, as noted by the authors. This should be amended.

Finally,NASH-specific clinical data (IHC or similar) that supports the model system would greatly increase the impact of the work.

In summary, excellent work which challenges existing dogma and extends the field, but which has a few minor flaws in its interpretation and lacks direct clinical support for the pathway described.

Referee #2 (Comments on Novelty/Model System):

This is an interesting study showing effects of RIP3 in necroptotic cell death and liver injury/fibrosis. It builds from a recent Cell paper that explored this paradigm and uncovered an unexpected role of caspase 8 inhibition to promote the necroptotic pathway. The main problems in the paper are that its limited to in vivo work and thus many mechanisms are left to assumption due to lack of in vitro studies. I don't know your journal so well but based on the high impact factor, I suspect the paper would need substantially more in vitro mechanism to compete for space. Members of my research trainee team assisted in completion of this review.

Referee #2 (Remarks):

This is a very interesting work that supports the concept that RIP3 mediated necroptosis plays a role in the pathophysiology of NASH model and that RIP3 inhibition might have clinical benefits in management of patients with NASH. It also raises cautions about caspase inhibition therapies since it implies that caspase 8 inhibition could promote necroptosis. The largest concern is that the work is limited to in vivo studies (except for a small control experiment in vitro) and thus many mechanisms are based on assumption without definitive in vitro interventions to prove them. This is important since prior studies have suggested that caspases and apoptosis are promoting liver injury and thus the data regarding caspase 8 are challenging the dogma in some ways. This is good but requires adequate proof. Many other smaller comments are outlined below many of which could be addressed by properly conducted and complementary in vitro studies.
1- There are no Fig. numbers for the Fig. section.
2- Authors are showing that caspase 8 deletion makes liver more susceptible to necroptosis shown by activation of RIP3 in MCD diet model in NASH. It would be important to show this in another model of fatty liver disease to confirm the findings. The MCD diet does not recapitulate human disease since mice lose weight unlike the human disease.
3- In Fig 2 S. It would be better to measure the levels of caspase 8 and cleaved caspase 3 in the same blots. Also these western blots in Fig 2S and Fig 1S are important and should probably be moved to the actual figure section.
4- Fig 2S and 8S change "expression" to "protein levels"
5- Authors mention that there is more fat accumulation in the Caspase 8 /RIP3 double KO mice. This is an interesting finding. How do they explain this finding?
6- In result section, first paragraph in the last 2 lines authors conclude that "while Caspase-8 in LPC suppresses RIP3-dependent liver injury in this model". This should probably be concluded after later experiments including the western blots done in Fig 2S.
7- What was the thought process of checking the markers of ER stress relative to RIP3 pathway? This is not well described in the result section 2nd paragraph, line 8.
8- Authors should also briefly discuss ER stress in connection to necroptosis and briefly mention the

role of EIF2 in ER stress in the introduction section. The ER stress angle is tangential and not that convincing. EIF2 can be phosphorylated by multiple kinases and CHOP can be induced by other mechanisms.
9- Fig 1C middle panel. Is there any significance in cleaved caspase 3, between casp 8 KO and other groups? This activation is not related to casp 8 pathway and authors conclude that it could be related to mitochondrial pathways? Is there any proof of mitochondrial injury or any other explanations for this? This should be moved to the supplementary section if no statistical significance. Also increase in cleaved caspase 3 does not necessarily mean there is more caspase 3 activity.
10- Page 7 second paragraph last 2 lines, "These data indicate that RIP3-dependent necroptosis promotes NASH-induced liver fibrosis. Moreover, activation of Caspase-8 inhibits RIP3-dependent liver fibrosis in NASH." How can they prove that this inhibition is caspase 8 dependent.
11- Page 8 line 1 , "response necroptosis" should be changed to "response to necroptosis"
12- It is known that inhibitors including JNK inhibitors have nonspecific targets. The authors have only used JNK inhibitor SP600125 in their experiments and concluded that JNK is downstream of the RIP3 pathway. It is important to inhibit JNK with another method eg. Antisense, etc to confirm their findings. In fact, JNK is well known to mediate liver injury in MCD diet and the observed effects with the inhibitor may not be related to RIPK3 activation.
13- The p-JNK is shown to be decreased in the western blot in Fig 5A in RIP3 KO and the double KO mice. This could be simply explained by the fact that these mice have less inflammation (fig3A) and less liver injury(Fig 1A) after MCD diet. How can the authors prove that JNK is actually downstream of casp 8 and RIP3 in their experiments?
14- Fig 5F . Does JNK inhibition have any effect on casp 8 on western blot? Like a loop mechanism?
15- The inflammation is more pronounced in the Casp 8 KO mice shown in Fig 3A and these mice have higher level of RIP3. Couldn't this increase be explained by more RIP3 due to more migration of inflammatory cells in the liver? And less RIP3 in double KO and RIP3 KO could be explained by the fact that they have less inflammation shown in Fig 3A.
16- In Fig 2S , ER stress markers are more pronounced in RIP3 KO and double KO on MCD diet comparing to WT on MCD but these mice have less ALT levels compared to WT (Fig 1A). How do you explain that?
17- Inclusion of TUNEL stain to complement cleaved caspase 3 stain would be more convincing to show hepatocyte cell death.

Referee #3 (Comments on Novelty/Model System):

MCD feeding is not appropriate as a NAS model, high-fat feeding and obesity mouse models are better models.

Not clear why LPC-caspase8-deficent mice on MCD had elevated liver injury than WT fed MCD. In previous report by Hatting shows that MCD feeding of WT caused injury and hepatocyte-caspase8 deficiency protects.

Referee #3 (Remarks):

This manuscript reports the role of RIP3-signalling in necroptosis in LPC-specific caspase8-deficient mice fed with MCD diet at 2 and 8 weeks. By cross-breeding LPC-specific caspase8-hets with RIP3-hets, the double knockout mice showed decreased liver injury, inflammation, fibrosis, and biliary ductular reaction. By administration of JNK inhibitor SP600125 to LPC-specific caspase8-deficient mice fed with MCD diet, protection against fibrosis and inflammation was observed, and associated with an inhibition of RIP3 protein indicating a feed-back loop RIP3 regulation. Authors concluded that RIP3 mediates liver injury, inflammation, fibrosis,..in MCD-induced NASH, and suggested that RIP3-dependent necroptosis may be used as a target for treatment of NASH.
1. The reviewer noticed that, when compared with chow, MCD feeding in WT mice caused some elevation of transaminases (Fig.1A), Ki67+ cells (Fig. 1C), fibrosis markers (Fig.2), and inflammation, particularly MCP-1 mRNA (Fig.3), and that RIP3 deficiency decreased these parameters. These observations indicate a cytotoxic role of RIP3 in liver injury independent of

caspase8. This raises a question whether RIP3 is really specific for necroptosis, and whether RIP3 is regulated by inflammation (while inflammation and necrotic cell death can be the cause of the other under in vivo conditions). Under certain conditions (cIAP1/2 deletion or TAK1 kinase inhibition, see Cell death Differ 20: 1381, 2013), RIP3 can participate in apoptosis, and can promote NLRP3 inflammasome activation in dendritic cells (Moriwaki and Chan Cytokine&Growth Factor Reviews 2014). Hence, RIP3 may not appropriately be considered as the necrosis-regulating molecule. Unfortunately (because the research field is relatively new), there are no effector molecules (distal from RIP3) which would specifically trigger necroptosis (Wallach D. et al. in Cytokine&Growth Factor Reviews 2014). While the observed protection in double knockouts under MCD NASH indicate the role of RIP3 in hepatic injury (not necessary necroapoptosis) in vivo, these data indeed confirm previous results during anti-Fas-induced hepatitis in caspase8 and RIP3 double knockouts (Kaiser W. et al. Nature 471: 368, 2011).
2. The latter notion raises a whether MCD model is appropriate as a NASH model. The protection by RIP3 deficiency (alone or combined with capase8 deficiency) was observed after 2 and 8 week MCD feeding. The reviewer noticed that WT mice at 8-week MCD feeding expressed increased hepatic MCP-1 mRNA compared to normal chow or 2-week MCD feeding (Fig. 3C). This indicates that MCD feeding in WT may induce liver inflammation (e.g., MCP-1) over time which can be even greater than that of MCD feeding of LPC-caspase8 knockout. There appears to be a time-course in terms of liver injury among feeding WT and LPC-caspase8-KO mice. Work form the same institution showed that MCD feeding for 10 weeks caused exacerbated liver injury, and protection could be obtained by hepatocyte-specific caspase8 deficiency (Hatting M et al. Hepatology 2013). These contradicting results were discussed by authors. While an alb-cre/Caspase-8 model was used in Hepatology 2013, the authors used the alfp-cre line in present study. The authors believe that that long-term MCD treatment in alb-cre/Caspase-8 mice promoted counterselection and inefficient Caspase-8 deletion in parenchymal liver cells in vivo, thus masked protective functions of Caspase-8 in NASH. As both experiments were performed by the same group or institution, please show the data and knockdown efficiency using the different mouse models for Caspase-8 deletion.
3. In MCD model, steatosis arises from increased fatty acid uptake and inhibition of VLDL secretion, and this diet causes lower liver phosphatidylcholine/phosphatidylethanolamine (PC/PE) ratio, rather than high PC/PE as reported in OB/OB or high-fat fed livers. MCD-fed mice actually lose body weights, thus does not represent typical NASH in humans. However, MCD feeding causes significant liver atrophy by which RIP3 here is shown to play a role in inflammation, and this is true for death-receptor hepatitis. This can also be seen that the protection by RIP3 deficiency was associated with further increased liver TG, rather than an inhibition (Fig. 2A, B). Hence, RIP3 deficiency protects inflammatory pathways rather than hepatic steatosis, and may not be effective in all NASH parameters hence RIP3 cannot represent a good target.
4. JNK activation occurs during lipotoxicity, apoptosis and inflammation. JNK as a downstream mediator by these major pathways may be a universal target for treatment of NASH. As shown in Fig. 5 that JNK inhibitor in LPC-caspase8 KO could inhibit RIP3 protein expression, in this context where does JNK lie relative to RIP3 and ER stress ? Mechanisms for this were not discussed.
5. The authors hypothesize that - although the RIP3 mouse used in this study is a full-body knockout model - the effects of RIP3 deficiency upon NASH are mainly hepatoparenchymal-derived. Based on Supplemental Figure 6, if the hepatoparenchymal cells really do mediate the RIP3-dependent effects upon NASH, then one should show it using a hepatoprenchymal cell-specific knockdown system, e.g. AAV-virus deliver system. Using a full-body knockout model instead of a cell type-specific knockout model, results in the danger of introducing bias from effects of extrahepatic organs (abdominal/ingiunal fat, brown adipose tissue etc.)

1st Revision - authors' response 15 April 2014

Reviewer #1:

We thank Reviewer #1 for his fair and professional evaluation of our manuscript. We are very grateful that Reviewer #1 acknowledges the quality and novelty of our study. We are convinced that the constructive comments of Reviewer #1 have led to a significant improvement of our manuscript.

*Comments of Reviewer #1:*

Ad 1: Reviewer #1 asks: *"In figure 3, MCP1 expression is not impacted by loss of C8 expression - though one would expect the difference seen at 2w to continue."*

We thank Reviewer #1 for this important comment referring to the lack of increase of MCP-1 mRNA levels in Casp-$8^{LPC-KO}$ mice fed 8 weeks with MCD-diet in comparison to WT mice. Considering that mRNA levels do not always reflect protein expression levels, we have now evaluated the intrahepatic protein levels of MCP-1 by using FACS-based micro-beads fluorescence assay. These experiments revealed a significant increase of MCP-1 levels after 8 weeks of MCD-diet feeding in Casp-$8^{LPC-KO}$ mice when compared to WT controls. Moreover, the respective MCP-1 levels in Casp-$8^{LPC-KO}$/RIP$3^{-/-}$ and RIP$3^{-/-}$ mice were dramatically reduced, thus further underlining the notion that MCP-1 represents an important factor linking RIP3-dependent necroptosis and chronic liver injury in NASH, as stated in the initial version of manuscript. Importantly, in Casp-$8^{LPC-KO}$/RIP$3^{-/-}$ and RIP$3^{-/-}$ mice, the levels of MCP-1 were comparable to those seen after 2 weeks of MCD-diet feeding although a slight increase in mRNA levels was observed. Together, these new findings strongly support our hypothesis that MCP-1 represents an important factor linking chronic liver injury and the presence of hepatic fibrosis in NASH. These experiment have now been added into the new Figure 3D.

Ad 2: Reviewer #1 asks: *"Loss of caspase-8 does increase CD45 recruitment and has been linked to inflammation in other tissues, such as skin. Is MCP1 really implicated in the process?"*

This important question goes into the same direction as the previous comment by Reviewer #1. We have now performed major additional work and are thus able to fully answer to this reviewer´s comment. Our new set of experiments on the expression of MCP-1 after 2 and 8 weeks of MCD-diet feeding in the different mouse stains demonstrated a close and significant correlation between MCP-1 expression levels and an increase of CD45+ and F4/80+ cells in injured livers. Of note, these findings are in line with several reports providing strong evidence that MCP-1 represents an important factor linking the recruitment of macrophages and the presence of liver fibrosis in chronic liver injury (Baeck et al., Gut 2012; 61:416-26; Seki et al., Hepatology 2009 50:185-97), which we stated in the text.

Ad 3: Reviewer #1 asks: *"Did the authors look at a 'gold standard' cytokine as a comparator, such as TNFa? This is particularly important, as there are reports that TNFa secretion is critically dependent upon RIPK3 - and central to the induction of necroptosis. Its effects on the monocyte compartment are well known."*

We thank Reviewer #1 for this very important question raised. To further clarify the role of TNF-a in our model we have performed additional experiments and analysed TNF-a concentrations by using qRT-PCR analysis on liver extracts from the different experimental groups of mice. Of note, these experiments revealed elevated levels of TNF-a especially after 8 weeks of MCD diet treatment in WT and Casp-$8^{LPC-KO}$ mice compared to the other experimental groups (New Supporting information Fig S7). In addition, a very recent publication has shown that TGF-β-receptor-II-dependent signals control hepatocyte cell death in NASH by controlling typical TNF-a-related signalling molecules like TAK1, NF-kB and ROS (Yang et al., Hepatology 2014; 59:483-95). Based on this, we tested levels of TGF-β2 in our groups and found a strong elevation in Casp-$8^{LPC-KO}$ mice after 2 and 8 weeks of MCD-diet feeding, which correlated to the massive expression of RIP3 in hepatocytes, while TGF-β2 levels were markedly decreased in Casp-$8^{LPC-KO}$/RIP$3^{-/-}$ and RIP$3^{-/-}$ mice. This important finding has been added into the new Supporting Information Fig S7.

Accordingly, this important point including the new reference has also been added in the main text as follows (page 9):

"*Further analyses revealed elevated TNF levels in WT and Casp-8$^{LPC-KO}$ mice after 8 weeks of MCD feeding, as well as a strong correlation between RIP3 expression levels and levels of TGF-β2 (Supporting Information Fig S7), which is in line with a recent report showing a prominent role of TGF-β in the regulation of NASH-associated hepatocyte cell death (Yang et al, 2014).*"

Ad 4: Reviewer #1 states: *"The authors refer to RIP3 or Casp-8 activation, but they never provide any evidence that catalytic activity of either enzyme is required for the phenotypes show. In fact, it is linked only to relative expression levels in these studies, and therefore a reader would not be able to link catalytic activity as 'required' in the models or not. In the absence of additional evidence, the text should be amended to indicate expression, rather than activity."*

We acknowledge this important comment of Reviewer #1, which is going into the same direction as the comment number #1 of Reviewer #3. However, to our best knowledge, there are not appropriate tools such as phospho-specific antibodies to directly analyze the activation of RIP3. Thus, in accordance with the comment raised by Reviewer #1, we have revised the text appropriately and agree that these changes will make our manuscript clearer to the reader.

Ad 5: Reviewer #1 asks: *"The lack of effect in the immune compartment of the cells at the mRNA level is interesting - the lack of arginase production (and TNFα) after IFNγ treatment of the RIP3 -/- appears to be significant. However, since these studies were done in RIP3 -/- mice, and not the (more correct) Casp8 LPC/ RIP3-/- (which may be different), the results remain supportive but not conclusive. Does a survey of proteins (via whatever means) grossly suggest concordance with mRNA levels? "*

Reviewer #1 raises the important point that our previous experiments were performed in RIP3$^{-/-}$ mice rather than the (more correct) Casp8$^{LPC-KO}$/RIP3$^{-/-}$ mice. Based on this comment, we have re-performed this experiment now including all different genotypes used in this study. Moreover, given the previous comments on possible differences between RNA and protein levels, we now used the cytometric bead assay technique to directly measure levels of inflammatory mediators in the cell culture supernatants (New Supporting Information Fig S8). As demonstrated in this comprehensive analysis, cultured macrophages isolated from mice of all genotypes had a very similar inflammatory response. Also morphologically, we could not see any difference between these cells.

We think that this is an important general finding for the interpretation of our study but also previous studies working with RIP3$^{-/-}$ mice in disease models. We are therefore very grateful for this comment of Reviewer #1, helping us to clarify this important point.

We have addressed this in the new text as follows (page 9):

"*However, in order to exclude that constitutive deletion of Rip3 in Casp-8$^{LPC-KO}$/RIP3$^{-/-}$ mice resulted in general signalling defects of immune cells as a reason for the rescue of these double-mutant animals from hepatic fibrosis, we isolated and cultured monocytes from murine bone marrow from WT, Casp-8$^{LPC-KO}$, Casp-8$^{LPC-KO}$/RIP3$^{-/-}$ and RIP3$^{-/-}$ mice.*"

Finally, a recent publication (Weinlich R et al., Cell Reports 2013; 5: 340-348) has also examined the functionality of Macrophages from RIP3$^{-/-}$ mice. In line with our present findings, they did not find significant differences in the activation capacity of these compared to WT macrophages.

Ad 6: Reviewer #1 states: *"In the final figure, the authors look at phospho-protein expression, but of course this data is not interpretable without control blots showing the total of each protein loaded (ie., JNK, p38, etc...)."*

We agree with Reviewer #1 and we have added the control blots showing the total of each protein loaded into the new Figure 6A.

Ad 7: Reviewer #1 states: *"It should be noted that the effect of systemic administration of a JNK inhibitor may well be on the bone marrow rather than the liver, so it is not clear that the pathway is critically involved in the liver, as noted by the authors. This should be amended."*

This comment of Reviewer #1 goes into a similar direction as comments #12/13 of Reviewer #2. As the Reviewer #1 remarks, we have stated in our initial manuscript that, by using a chemical JNK-inhibitor, we could not exclude that JNK acts also in non-parenchymal cells (bone marrow cells or Kupffer cells) and we had referred to the previous literature, e.g. the study by Das et al. (Page 14 of the discussion section). Given that both Reviewers had referred to this, we aimed to provide further functional information on this question and performed a new set of experiment in L929 cells, which are very prone to necroptosis development even by application of apoptosis inhibitors (Vercammen D et al., J Exp Med 1998; 187:1477–1485). We could confirm that stimulation of these cells with zVAD induced necroptosis (RIP3 up regulation and cell death). This process could not only be inhibited by the necroptosis-inhibitor Nec-1, but also the JNK inhibitor SP-600125 (New Supporting Information Fig S10), which importantly went along with reduced RIP3 levels in these cells. These findings support the hypothesis that the reduction in cell death and RIP3 expression levels found in our mouse livers treated with SP600125 was not only related to bone marrow cells, but also by affecting directly signalling processes in hepatocytes.

These new findings have been summarized in the New Supporting Information Fig. S10 and also were addressed in the manuscript text as follows :

Page 11:

"*To further confirm a mutual interaction between RIP3 and JNK signalling, we used L929 cells and confirmed that these cells undergo necroptosis upon stimulation with the pan-Caspase-inhibitor zVAD (Supporting Information Fig S10). Of note, additional treatment with the necroptosis inhibitor Nec-1 (Degterev et al, 2013) and also with SP600125 abolished zVAD-induced cell death. Moreover, JNK-inhibition was associated with reduced RIP3 expression levels (Supporting Information Fig S10). These data suggest that activation of JNK in LPC and probably non-parenchymal cells (NPC) further augments hepatic RIP3 signalling in terms of a positive feedback loop."*

Pages 14/15:

*"However, our experiments in L929 cells suggest that a cell-autonomous or intercellular feedback loop exists in hepatocytes between RIP3- and JNK-signalling."*

Ad 8: Reviewer #1 states: *"Finally, NASH-specific clinical data (IHC or similar) that supports the model system would greatly increase the impact of the work."*

We recognize this as the most important comment raised by the referees and the editor. Based on this important comment we have performed a completely new analysis of RIP3 expression in human

NASH patients. Fortunately, we were able to collaborate with two major clinical centres for the treatment of NASH patients, Dr. Helen Reeves (Newcastle/GB) and Dr. Ali Canbay (Essen University/Germany). These centres provided us with biopsy tissue and paraffin sections of independent, well-characterized cohorts of NASH patients treated at their centres, on which we could perform a comprehensive analysis of RIP3 expression (immunohistochemistry and Western blot). Both analyses revealed a clear and consistent up-regulation of RIP3 expression in NASH patients. These findings strongly support the relevance of our findings in mouse models for a possible translation into the clinics. Given the importance of these findings for our paper, we included them as a new main Figure into the manuscript (New Figure 5).

We addressed this new figure in the main text as follows (pages 10/11):

"*It was previously demonstrated in liver samples from human NASH patients that RIP3 is strongly upregulated on RNA-level to more than 40 fold compared to healthy controls (Csak et al, 2011). In order to provide further evidence for a function of RIP3 in human NASH, we examined RIP3 expression in livers of NASH patients (as demonstrated histologically by elevated NAS score [Fig 5A]) by Western blot and immunohistochemistry. On protein levels, RIP3 was strongly upregulated in NASH patients compared to controls (Fig.5B). Immunostaining of NASH patient livers revealed strong RIP3 expression in hepatocytes, often neighbouring areas of fat deposition (Fig 5C). Of note, RIP3 often showed a granule-like staining pattern (Fig 5C), similar to previous imaging results in MEF cells with activated RIP3 signalling depecting clustering of RIP1/RIP3 (Li J et al, 2012). Finally, RIP3 was often overexpressed in cells morphologically reflecting cholangiocytes / bile duct cells (Fig 5C), similar our previous findings in mouse livers. These findings support the hypothesis that also in human NASH, liver cells are sensitized to necroptotic cell death. Moreover, in murine as well as human NASH, biliary cells express high levels of RIP3, pointing towards cell-type specific functions of this pathway in the liver.*"

Reviewer #2:

We thank Reviewer #2 for his fair and detailed evaluation of our manuscript. Based on his helpful comments we have taken major efforts in order to provide further experiments to strengthen the mechanistic aspects on the role of necroptosis in the development of NASH.

*Comments of Reviewer #2:*

Ad 1: Reviewer #2 states: *"There are no Fig. numbers for the Fig. section."*

We are grateful to Reviewer #2 for the careful reading of our manuscript and apologize for this mistake. In the revised version of the manuscript the Fig. numbers have been added.

Ad 2: Reviewer #2 states: *"Authors are showing that caspase 8 deletion makes liver more susceptible to necroptosis shown by activation of RIP3 in MCD diet model in NASH. It would be important to show this in another model of fatty liver disease to confirm the findings. The MCD diet does not recapitulate human disease since mice lose weight unlike the human disease."*

We are thankful to the reviewer for this valuable comment regarding the ability to reflect molecular processes occurring during human NASH. We clearly acknowledge that all available rodent models of chronic liver injury and fatty liver disease have certain drawbacks. Although high fat diet (HFD)

increases body weight and induces insulin resistance that represent important features of human NAFLD; HFD-induced liver fat accumulation may not follow a linear progression and liver fat levels may actually decrease (Gauthier MS et al., Br J Nutr 2006; 95:273-81). More importantly, we picked this model since to our knowledge it best recapitulates the transition from fatty liver disease (NAFLD) towards inflammation (NASH) and liver fibrosis, which, as stated in the introduction, represents the primary determinant of mortality and probably the most important end point in human NASH studies. In contrast, pure high fat diet models might reflect important metabolic features of human NASH, but are rarely associated with fibrosis. That is why many previous experimental studies on the specific role of cell death and apoptosis in NASH applied the MCD model and these findings were even the basis for clinical studies on apoptosis inhibitors. Based on this, in order to highlight the differential functions of necroptosis and apoptosis, we used this respective model in our present study.

However, in accordance with this important reviewer's comment and also to take into consideration comment number #3 of Reviewer #3, we performed major additional work to show that this function of RIP3 is specific to metabolic liver injury and not a general reaction to cytotoxic agents, and applied the highly standardized model of repetitive injection of carbon tetrachloride (2 weeks and 6 weeks). These new experiments revealed no differences in collagen deposition between all different experimental groups, as demonstrated in the New Supporting Information Fig S4, supporting a specific role of RIP3 in fatty-liver related injury and fibrosis.

This finding has been addressed in the main text as follows (Page 7/8):

*"We have further addressed the question if the previously shown pro-fibrogenic effect of RIP3 is specific for liver fibrosis in response to hepatic steatosis or represents a general principle in hepatic fibrogenesis. To test this, we used and alternative, very well established model of experimental liver fibrosis relying on repetitive injections of the substance $CCl_4$ into mice and applied this model for 2 and 6 weeks to WT, Casp-8$^{LPC\text{-}KO}$, Casp-8$^{LPC\text{-}KO}$/RIP3$^{-/-}$ and RIP3$^{-/-}$ mice. This treatment led to the development of areas of parenchymal cell necrosis in Casp-8$^{LPC\text{-}KO}$ mice (Supporting Information Fig S4). However, it did not result in a significantly increased degree of fibrosis between the groups of mice in quantitative analysis of Sirius Red staining (Supporting Information Fig S4), supporting the hypothesis that RIP3 might represent a specific target in fatty-liver related liver fibrosis."*

Ad 3: Reviewer #2 states: *"In Fig 2 S. It would be better to measure the levels of caspase 8 and cleaved caspase 3 in the same blots. Also these western blots in Fig 2S and Fig 1S are important and should probably be moved to the actual figure section."*

We thank Reviewer #2 for this important comment. We have now added analyses on Caspase-8 expression together with RIP3 into the main Figure 1B.

Ad 4: Reviewer #2 states: *"*Fig 2S and 8S change "expression" to "protein levels"".

We thank Reviewer #2 for carefully reading our manuscript. We have corrected the word "expression" by "protein levels" accordingly.

Ad 5: Reviewer #2 states: *"*Authors mention that there is more fat accumulation in the Caspase-8/RIP3 double KO mice. This is an interesting finding. How do they explain this finding?"

Reviewer #2 makes an important comment addressing a part of our discussion section in which we had discussed if the occurrence of different programmed cell deaths might influence the degree of steatosis. It appears that the blockage of both cell death pathways – apoptosis and necroptosis – makes hepatocytes more tolerant to fat accumulation, suggesting that these cell death pathways may act as sensors for hepatic fat accumulation. Moreover, given that apoptosis and necroptosis might regulate others programmed cell death pathways such as autophagy or other pathways involved in lipolysis, these might be altered in Casp-8$^{LPC-KO}$/RIP3$^{-/-}$ mice (Liu & Czaja, 2013).

We discussed this finding in our manuscript as follows (page 14):

*"Instead, blockage of both cell death pathways (necroptosis and Caspase-8-dependent apoptosis) in Casp-8$^{LPC-KO}$/RIP3$^{-/-}$ mice resulted in an increase in intrahepatic fat accumulation in this model compared with WT or single mutant animals, suggesting that absence of these two programmed cell death pathways might increase the tolerance of hepatocytes to store lipids without undergoing cell death. Alternatively, given that multiple molecular interactions between programmed cell death pathways and autophagy have been suggested (Pattingre et al, 2005; Yousefi et al, 2006), simultaneous inhibition of Caspase-8-dependent apoptosis and necroptosis might alter the activity of cellular pathways controlling lipolysis in hepatocytes (Liu & Czaja, 2013)."*

Ad 6: Reviewer #2 states: *"In result section, first paragraph in the last 2 lines authors conclude that "while Caspase-8 in LPC suppresses RIP3-dependent liver injury in this model". This should probably be concluded after later experiments including the western blots done in Fig 2S."*

As requested by Reviewer #2, we have removed this statement from that early part of the manuscript. Moreover, as requested, we verified abrogation of Caspase-8 expression in Casp-8$^{LPC-KO}$ and Casp-8$^{LPC-KO}$/RIP3$^{-/-}$ mice by Western blot and added this into the new Figure 1B.

Ad 7: Reviewer #2 states: *"What was the thought process of checking the markers of ER stress relative to RIP3 pathway? This is not well described in the result section 2nd paragraph, line 8."*

Ad 8: Reviewer #2 states: *"Authors should also briefly discuss ER stress in connection to necroptosis and briefly mention the role of EIF2 in ER stress in the introduction section. The ER stress angle is tangential and not that convincing. EIF2 can be phosphorylated by multiple kinases and CHOP can be induced by other mechanisms."*

We thank Reviewer #2 for pointing these important comments that helped us to further focus our manuscript. For the initial version of our manuscript, we decided to check markers of ER-stress for two reasons. First, ER-stress is linked to the development of NASH (Gentile et al., J Nutr Biochem 2008; 19:567-76) and second, it is related to JNK-mediated cell death (Kyriakis JM et al., Nature 1994; 369:156-60). However, we agree with Reviewer #2 that ER-stress part is not well connected to the other parts of the manuscript and may distract the reader from our main messages. To best accomplish with these referee's comments, we decided to remove the ER stress part from the revised version of the manuscript. We are planning to analyze the relation between programmed cell death pathways and ER stress in a new study, but we agree with Reviewer #2 that this is out of scope from the present study. Therefore, we thank the referee for this important comment that helped us to make the paper much more concise and less speculative.

Ad 9: Reviewer #2 states: *"Fig 1C middle panel. Is there any significance in cleaved caspase 3, between casp 8 KO and other groups? This activation is not related to casp 8 pathway and authors conclude that it could be related to mitochondrial pathways? Is there any proof of mitochondrial*

*injury or any other explanations for this? This should be moved to the supplementary section if no statistical significance. Also increase in cleaved caspase 3 does not necessarily mean there is more caspase 3 activity."*

We thank Reviewer #2 for this important comment regarding the activation of apoptosis in our mouse model of NASH. As shown in the New Supplemental Figure S2, a very low level of apoptosis is detected in Casp-8$^{LPC-KO}$ mice that is not significantly higher in comparison to the others groups of mice. These data suggest the contribution of Caspase-8 independent pathways for triggering apoptosis in response to MCD-diet feeding. Indeed, as suspected, there is indication in literature that fatty acid accumulation enhances b-oxidation consequently electron overflow in the mitochondrial electron transfer chain, triggering cell death (Seifert EL et al., J Biol Chem 2010; 285:5748-5758.). We have now added this important citation into our manuscript to be less speculative regarding this point.

Caspase-3 is activated by cleavage (Nicholson DW et al., Trends Biochem Sci 1997; 22:299-306) and is often used as biomarker for apoptosis. Nevertheless, according to the comment of Reviewer #2, we have changed into the manuscript "activity" by "cleavage".

Ad 10: Reviewer #2 states: *"Page 7 second paragraph last 2 lines, "These data indicate that RIP3-dependent necroptosis promotes NASH-induced liver fibrosis. Moreover, activation of Caspase-8 inhibits RIP3-dependent liver fibrosis in NASH." How can they prove that this inhibition is caspase 8 dependent."*

It is known that Caspase-8 inhibits RIP3-dependent necroptosis by cleaving RIP3 (Feng S et al., Cell Signal 2007; 19:2056-67). We describe a strong increase of liver injury in absence of Caspase-8, which coincides with RIP3 over-expression. This phenotype can be rescued by additional deletion of RIP3. All together, these genetic experiments provide strong evidence that Caspase-8 inhibits RIP3-dependent liver fibrosis in the present model.

Ad 11: Reviewer #2 states: *"Page 8 line 1, "response necroptosis" should be changed to "response to necroptosis""*

We are grateful to Reviewer #2 for carefully reading of our manuscript. We have corrected this mistake.

Ad 12/13: Reviewer #2 states: *"It is known that inhibitors including JNK inhibitors have nonspecific targets. The authors have only used JNK inhibitor SP600125 in their experiments and concluded that JNK is downstream of the RIP3 pathway. It is important to inhibit JNK with another method e.g. Antisense, etc. to confirm their findings. In fact, JNK is well known to mediate liver injury in MCD diet and the observed effects with the inhibitor may not be related to RIPK3 activation."*

Moreover, Reviewer #2 states: *"The p-JNK is shown to be decreased in the western blot in Fig 5A in RIP3 KO and the double KO mice. This could be simply explained by the fact that these mice have less inflammation (fig3A) and less liver injury (Fig 1A) after MCD diet. How can the authors prove that JNK is actually downstream of casp 8 and RIP3 in their experiments?"*

These important comments of Reviewer #2 go into a similar direction as comment number #7 of Reviewer #1. As stated in the initial version of our manuscript, we also believe that JNK is not only

downstream of RIP3, but we rather provide evidence that a mutual interaction exists between JNK and RIP3. We agree with Reviewer #2 that based on our experiments we could not exclude that JNK levels might be influenced by inflammation. Based on the valuable comment of Reviewer #2 as well as a similar comment by Reviewers #1 and #3, we decided to carefully investigate the relationship between RIP3 and JNK *in vitro* using L929 cells, representing an established model for studying necroptosis. Among the multitude of available caspase inhibitors, zVAD-fmk (zVAD) is probably the most commonly used pan-caspase inhibitor because of its direct inhibition of Caspase-8 and its capacity to induce strong necrotic cell death in L929 cells. As shown in the New Supplemental Figure S10, inhibition of JNK signalling with the pharmacological inhibitor SP600125 completely blocked zVAD-induced necroptosis in L929 cells to a similar extent as cells treated with Necrostatin-1 (Nec-1) that specifically blocks the kinase activity of RIP1 necessary for the induction of necroptosis. Interestingly, the inhibition of JNK went along with reduced protein levels of RIP3, supporting the relationship between RIP3 and JNK in a mono-cellular in vitro system. We believe that these findings go into the same direction as and corroborate our *in vivo* data, showing the reduction of intra-hepatic RIP3 levels in Casp-8$^{LPC-KO}$ mice fed 2 weeks with MCD-diet and treated with the inhibitor SP600125.

We have included these important findings into the main text as follows (page 12): "*To further confirm a mutual interaction between RIP3 and JNK signalling, we used L929 cells and confirmed that these cells undergo necroptosis upon stimulation with the pan-Caspase-inhibitor zVAD (Supporting Information Fig S10). Of note, additional treatment with the necroptosis inhibitor Nec-1 (Degterev et al, 2013) and also with SP600125 abolished zVAD-induced cell death. Moreover, JNK-inhibition was associated with reduced RIP3 expression levels (Supporting Information Fig S10). These data suggest that activation of JNK in LPC and probably non-parenchymal cells (NPC) further augments hepatic RIP3 signalling in terms of a positive feedback loop.*"

Ad 14: Reviewer #2 states: *"Fig 5F . Does JNK inhibition have any effect on casp 8 on western blot? Like a loop mechanism?"*

In the respective Figure 5F, we had used Casp-8$^{LPC-KO}$ mice. The mice were fed 2 weeks with MCD-diet; one group was treated with DMSO and the other one with the JNK inhibitor SP600125. These experiments revealed that the hepatic expression of RIP3 is influenced by the administration of JNK inhibitor and were already included in the initial version of the manuscript. As shown in Figure 1B, there is hardly any expression of Caspase-8 in Casp-8$^{LPC-KO}$ mice. Given the Referee's comment, we believe that this fact was not illustrated clearly enough in the initial version of the manuscript. For the revised version of the manuscript we have modified the figure accordingly in order to provide a highest level of clarity.

Ad 15: Reviewer #2 states: *"The inflammation is more pronounced in the Casp 8 KO mice shown in Fig 3A and these mice have higher level of RIP3. Couldn't this increase be explained by more RIP3 due to more migration of inflammatory cells in the liver? And less RIP3 in double KO and RIP3 KO could be explained by the fact that they have less inflammation shown in Fig 3A."*

We thank Reviewer #2 for the important comment referring to the inflammation that could increase RIP3 expression in Casp-8$^{LPC-KO}$ after MCD-diet feeding. In our recent paper published in Cell Reports (Vucur M et al., Cell Rep 2013; 4:776-90), we had thoroughly examined the spontaneous phenotype of untreated Casp-8$^{LPC-KO}$ animals. As corroborated in the present study, we had shown in the previously published paper that Casp-8$^{LPC-KO}$ mice show increased expression of RIP3 in basal conditions. However, they did not show any signs of increased inflammation with regards to intra-hepatic levels of immune cells (CD45$^{+}$ and F4/80$^{+}$ cells) compared to WT mice. These findings suggest a direct activation of necroptosis in Casp-8$^{LPC-KO}$ mice due to increased level of RIP3, independently of inflammation. Moreover, in the present study we confirmed our Western blot

analyses by immunostaining experiments, showing that RIP3 is clearly overexpressed in parenchymal liver cells (hepatocytes and cholangiocytes), which we could now also confirm in human livers from NASH patients.

Ad 16: Reviewer #2 states: *"In Fig 2S , ER stress markers are more pronounced in RIP3 KO and double KO on MCD diet comparing to WT on MCD but these mice have less ALT levels compared to WT (Fig 1A). How do you explain that?"*

As stated in our response to the previous comments #7 and #8, we removed the analyses on modulators of ER stress in favour of the manuscript's clarity.

Ad 17: Reviewer #2 states: *"Inclusion of TUNEL stain to complement cleaved caspase 3 stain would be more convincing to show hepatocyte cell death."*

Similarly to our immunohistochemical analysis of cl-Caspase-3, we could not detect any differences in TUNEL stain between the different experimental groups of mice (data not shown). Based on the editor comment, we did not include these results to the manuscript.

Reviewer #3:

We thank Reviewer #3 for his careful evaluation and his fair and thoughtful comments, which helped us to improve the quality of our manuscript. These are our detailed responses:

*Comments of Reviewer #3:*

Ad 1: Reviewer #3 states: *"The reviewer noticed that, when compared with chow, MCD feeding in WT mice caused some elevation of transaminases (Fig.1A), Ki67+ cells (Fig. 1C), fibrosis markers (Fig.2), and inflammation, particularly MCP-1 mRNA (Fig.3), and that RIP3 deficiency decreased these parameters. These observations indicate a cytotoxic role of RIP3 in liver injury independent of caspase8. This raises a question whether RIP3 is really specific for necroptosis, and whether RIP3 is regulated by inflammation (while inflammation and necrotic cell death can be the cause of the other under in vivo conditions). Under certain conditions (cIAP1/2 deletion or TAK1 kinase inhibition, see Cell death Differ 20: 1381, 2013), RIP3 can participate in apoptosis, and can promote NLRP3 inflammasome activation in dendritic cells (Moriwaki and Chan Cytokine&Growth Factor Reviews 2014). Hence, RIP3 may not appropriately be considered as the necrosis-regulating molecule. Unfortunately (because the research field is relatively new), there are no effector molecules (distal from RIP3) which would specifically trigger necroptosis (Wallach D. et al. in Cytokine&Growth Factor Reviews 2014). While the observed protection in double knockouts under MCD NASH indicate the role of RIP3 in hepatic injury (not necessary necroapoptosis) in vivo, these data indeed confirm previous results during anti-Fas-induced hepatitis in caspase8 and RIP3 double knockouts (Kaiser W. et al. Nature 471: 368, 2011)."*

Reviewer #3 is raising the point that RIP3 might not be specific for necroptosis since RIP3 has been shown to regulate apoptosis under certain genetic conditions, e.g. cIAP- and TAK1 inhibition. This is an important point. In line with this, we had critically discussed differences in the outcome of our own previous findings in TAK1 knockout mice (pages 16 and 17 in the text). However, in the previous studies on RIP3 knockout mice (without additional genetic defects like TAK1- or cIAP deletion) in disease models relying on stimulation with specific substances (e.g. the recent Nature Medicine Paper on a model of Gaucher's disease (Vitner EB et al., Nat Med 2014; 20:204-8) or the 2009 Cell Paper on a pancreatitis model relying on Cerulein injections (He S et al., Cell 2009; 137:1100-11), the authors interpreted a reduction of cell death in these mice as genetic evidence for necroptosis. However, based on the profound comment of Reviewer #3, we carefully re-evaluated if there might be any indication for increased apoptosis in RIP3-overexpressing Casp-8$^{LPC-KO}$ mice (Supporting Information Fig S2), but could not find any indication for this. We also confirmed the presence of TAK1 and cIAPs in livers of Casp-8$^{LPC-KO}$ mice (data not shown).

Ad 2: Reviewer #3 states: *"The latter notion raises a whether MCD model is appropriate as a NASH model. The protection by RIP3 deficiency (alone or combined with capase8 deficiency) was observed after 2 and 8 week MCD feeding. The reviewer noticed that WT mice at 8-week MCD feeding expressed increased hepatic MCP-1 mRNA compared to normal chow or 2-week MCD feeding (Fig. 3C). This indicates that MCD feeding in WT may induce liver inflammation (e.g., MCP-1) over time which can be even greater than that of MCD feeding of LPC-caspase8 knockout. There appears to be a time-course in terms of liver injury among feeding WT and LPC-caspase8-KO mice.*

We thank Reviewer #3 for this important comment. Reviewer #3 is right in that also WT mice develop hepatic inflammation in response to MCD feeding. Importantly, this inflammation seen in WT mice correlates with a mild increase in RIP3 expression. In Casp-8$^{LPC-KO}$ mice, both RIP3 expression (Figure 1B) and inflammation (MCP-1 levels, CD45$^{+}$ cells, macrophages) (Figure 3) are upregulated, supporting – from our point of view – the correlation between RIP3-dependent necroptosis and hepatic inflammation leading to liver fibrosis. We fully agree with the referee that there is a time course in terms of liver injury, since 8 weeks of MCD feeding in both groups resulted in clearly more fibrosis than 2 weeks [fibrotic areas in WT mice: 2 weeks =0.6% vs. 8 weeks =1,8%; in Casp-8$^{LPC-KO}$ mice: 2 weeks =2% vs. 8 weeks =3,9%; compare Figure 2 and Supporting Information Fig S3).

*Work form the same institution showed that MCD feeding for 10 weeks caused exacerbated liver injury, and protection could be obtained by hepatocyte-specific caspase8 deficiency (Hatting M et al. Hepatology 2013). These contradicting results were discussed by authors. While an alb-cre/Caspase-8 model was used in Hepatology 2013, the authors used the alfp-cre line in present study. The authors believe that that long-term MCD treatment in alb-cre/Caspase-8 mice promoted counterselection and inefficient Caspase-8 deletion in parenchymal liver cells in vivo, thus masked protective functions of Caspase-8 in NASH. As both experiments were performed by the same group or institution, please show the data and knockdown efficiency using the different mouse models for Caspase-8 deletion."*

This is an important comment. We would like to state that, although from the same institution, we are an independent research group and used completely different mouse models than in the previous study. To address this question, we could receive livers from albumin-cre/Caspase-8Floxed mice (non-treated) that were still available from the mentioned previous study. While under basal conditions we could not see differences in the deletion efficiency of Caspase-8, we saw clearly higher basal levels of RIP3 in our alfp-cre mice (which is in line with most previous studies in other organs using Caspase-8 knockout mice, e.g. the recent skin paper from Weinlich R et al. Cell Reports 2013 and with the fact that Caspase-8 can cleave RIP3 (Feng S et al., Cell Signal 2007; 19:2056-67) than their alb-cre mice (which is in line with most previous studies in other organs

using Caspase-8 knockout mice and with the fact that Caspase-8 can cleave RIP3 (Feng S et al., Cell Signal 2007; 19:2056-67). This finding is important, since it clearly supports the close correlation between liver injury and RIP3 overexpression in NASH. Therefore, we thank the reviewer #3 for this important comment helping us to characterize our model better and to be less speculative. The Western blot analyses of the different mouse cre-lines have been added as New Supporting Information Fig S11 and based of this important comment we have changed the text accordingly (Page 15):

*"Of note, comparison of RIP3-expression levels between alfp-cre/Caspase-8Fl and alb-cre/Caspase-8Fl confirmed high RIP3 expression upon alfp-cre-mediated Caspase-8 deletion (Supporting Information Fig S11), which is in line with previous reports on Caspase-8 deletion in other organs like skin (Weinlich R et al. Cell Reports 2013). In contrast, we did not detect RIP3 up-regulation upon albumin-cre-mediated deletion, further supporting the association between RIP3 expression levels and necroptotic liver injury."*

This finding is especially interesting given that the alfp-cre line contains an alpha-fetoprotein enhancer element allowing efficient deletion not only in hepatocytes but also biliary cells and precursor cells. Together with our new findings that RIP3 seems to be highly expressed in these respective compartments, this might underline the role of these cells in necroptotic liver injury and NASH. We will take the important comment of the referee for further analyses and plan to use other cre-lines to follow up on this. However, in order to avoid too much speculation in the present manuscript, we presently did not add this latter point into the discussion.

Ad 3: Reviewer #3 states: *"In MCD model, steatosis arises from increased fatty acid uptake and inhibition of VLDL secretion, and this diet causes lower liver phosphatidylcholine/ phosphatidylethanolamine (PC/PE) ratio, rather than high PC/PE as reported in OB/OB or high-fat fed livers. MCD-fed mice actually lose body weights, thus does not represent typical NASH in humans. However, MCD feeding causes significant liver atrophy by which RIP3 here is shown to play a role in inflammation, and this is true for death-receptor hepatitis. This can also be seen that the protection by RIP3 deficiency was associated with further increased liver TG, rather than an inhibition (Fig. 2A, B). Hence, RIP3 deficiency protects inflammatory pathways rather than hepatic steatosis, and may not be effective in all NASH parameters hence RIP3 cannot represent a good target."*

Reviewer #3 is addressing an important issue in validating the MCD model. We fully agree that there are some limitations in regards of MCD model but this model also mimics some very important aspects of human NASH, including the development of steatohepatitis, CYP2E1 overexpression and increased lipid peroxidation as well as the promotion of NASH towards hepatic fibrosis which is not seen in many others models of NASH such as high fat diet model. Most importantly, it reflects the sequence from NASH to fibrosis which is the main determinant of outcome of human NASH patients that is why many previous experimental studies on cell deaths in NASH have been performed using the MCD model.

In order to further address this important comment by the Reviewer #3, we decided to clarify the specificy of RIP3 signalling for fatty-liver-induced cell injury and inflammation by applying an alternative model for inflammatory liver fibrosis, the $CCl_4$ model relying on repetitive injections with the toxic substance carbon tetrachloride. These new experiments revealed no differences in collagen deposition between all different experimental groups, as demonstrated in the new Supporting Information Fig S4, supporting a specific role of RIP3 in fatty-liver related injury and fibrosis. This finding has been addressed in the main text as follows (Page 7/8):

*"We further addressed the question if the previously shown pro-fibrogenic effect of RIP3 is specific for liver fibrosis in response to hepatic steatosis or represents a general principle in hepatic*

*fibrogenesis. To test this, we used and alternative, very well established model of experimental liver fibrosis relying on repetitive injections of the substance $CCl_4$ into mice and applied this model for 2 and 6 weeks to WT, Casp-8$^{LPC-KO}$, Casp-8$^{LPC-KO}$/RIP3$^{-/-}$ and RIP3$^{-/-}$ mice. This treatment led to the development of areas of parenchymal cell necrosis in Casp-8$^{LPC-KO}$ mice (Supporting Information Fig S4). However, it did not result in a significantly increased degree of fibrosis between the groups of mice in quantitative analysis of Sirius Red staining (Supporting Information Fig S4), supporting the hypothesis that RIP3 might represent a specific target in fatty-liver related liver fibrosis."*

Ad 4: Reviewer #3 states: *"JNK activation occurs during lipotoxicity, apoptosis and inflammation. JNK as a downstream mediator by these major pathways may be a universal target for treatment of NASH. As shown in Fig. 5 that JNK inhibitor in LPC-caspase8 KO could inhibit RIP3 protein expression, in this context where does JNK lie relative to RIP3 and ER stress ? Mechanisms for this were not discussed."*

This important comment of Reviewer #3 goes into a similar direction as comment number #7 of Reviewer #1. As stated in the initial version of our manuscript, our findings suggested that a mutual interaction interaction exists between JNK and RIP3. Based on the valuable comment of Reviewer #3, we decided to carefully investigate the relationship between RIP3 and JNK in vitro using L929 cells, representing an established model for studying necroptosis. Among the multitude of available caspase inhibitors, zVAD-fmk (zVAD) is probably the most commonly used pan-caspase inhibitor because of its direct inhibition of Caspase-8 and its capacity to induce strong necrotic cell death in L929 cells. As shown in the new Supplemental Figure S10, inhibition of JNK signalling with the pharmacological inhibitor SP600125 completely blocked zVAD-induced necroptosis in L929 cells to a similar extent as cells treated with Necrostatin-1 (Nec-1) that specifically blocks the kinase activity of RIP1 necessary for the induction of necroptosis. Interestingly, the inhibition of JNK went along with reduced protein levels of RIP3, supporting the relationship between RIP3 and JNK in a mono-cellular in vitro system. We believe that these findings go into the same direction as our in vivo data, showing the reduction of intra-hepatic RIP3 levels in Casp-8$^{LPC-KO}$ mice fed 2 weeks with MCD-diet and treated with the inhibitor SP600125.

We have included these important findings into the main text as follows (page 12):

"*To further confirm a mutual interaction between RIP3 and JNK signalling, we used L929 cells and confirmed that these cells undergo necroptosis upon stimulation with the pan-Caspase-inhibitor zVAD (Supporting Information Fig S10). Of note, additional treatment with the necroptosis inhibitor Nec-1 (Degterev et al, 2013) and also with SP600125 abolished zVAD-induced cell death. Moreover, JNK-inhibition was associated with reduced RIP3 expression levels (Supporting Information Fig S10). These data suggest that activation of JNK in LPC and probably non-parenchymal cells (NPC) further augments hepatic RIP3 signalling in terms of a positive feedback loop*."

With regards to the second part of the Referee's comment, it is commonly believed that ER stress can activate JNK (Moretti et al., Cell Cycle 2007; 6:793-798). However, we also performed some Western blots suggesting that inhibition of JNK also led to reduced expression of ER stress related proteins like CHOP and Elf2 (data not shown). Again, we took the referee's important comment towards the unclear relationship between ER stress and JNK signalling as well as the previous comment numbers #7/8 from Reviewer #2 as reason to remove the analyses on ER stress markers from the manuscript. We agree that the functional relationship of ER stress with the current cell death pathways is probably more complex and out of scope of our manuscript, and taking it out made our manuscript more concise and clearer. Hence, we thank Reviewer #3 for this important comment.

Ad 5: Reviewer #3 states: *"The authors hypothesize that - although the RIP3 mouse used in this study is a full-body knockout model - the effects of RIP3 deficiency upon NASH are mainly hepatoparenchymal-derived. Based on Supplemental Figure 6, if the hepatoparenchymal cells really do mediate the RIP3-dependent effects upon NASH, then one should show it using a hepatoprenchymal cell-specific knockdown system, e.g. AAV-virus deliver system. Using a full-body knockout model instead of a cell type-specific knockout model, results in the danger of introducing bias from effects of extrahepatic organs (abdominal/ingiunal fat, brown adipose tissue etc.)"*

The question of cell specificity is an important point raised by the referee. As the referee states, it would have been ideal to use also liver-cell specific knockout mice in our study, but for RIP3, to our knowledge these do not exist. That is why we tried to control our experiments as well as possible.

1. In Casp-8$^{LPC-KO}$ mice (generated with a very well characterized cre-line that to our best knowledge only deletes in parenchymal liver cells) we show strong hepatic overexpression of RIP3 in parenchymal cells (hepatocytes, biliary cells), going along with elevated ALT levels (liver cell specific marker) which then leads to fibrosis. The fact that this phenotype could be fully restored by crossing with RIP3$^{-/-}$ mice provides strong evidence for a role of RIP3 in parenchymal liver cells.

2. The suggestion to use a viral delivery system to use in vivo in hepatocytes is per se interesting. However, it should be noted that in the initial papers characterizing necroptosis (Cho YS, Cell 2009, 137(6):1112-23), it was shown that there is a strong effect of RIP3 signalling in virus-induced liver injury that might most probably bias any *in vivo* results. Also, the combination of this treatment with long term MCD diet treatment appears problematic.

3. The referee is right that using a full knockout mouse of RIP3 cannot fully exclude an interaction of this pathway with other tissues than liver, e.g. fat tissue. Anticipating for immune cells, we had included in our initial study the control experiment on macrophages from RIP3$^{-/-}$ cells, that did not show any clear differences. A possible interaction with fat tissue in NASH is an interesting aspect that would be worth a further examination. At present, a comprehensive immunohistochemical analysis for immune-cell markers of white and brown fat tissue did not show any obvious differences between the different mouse groups (see below).

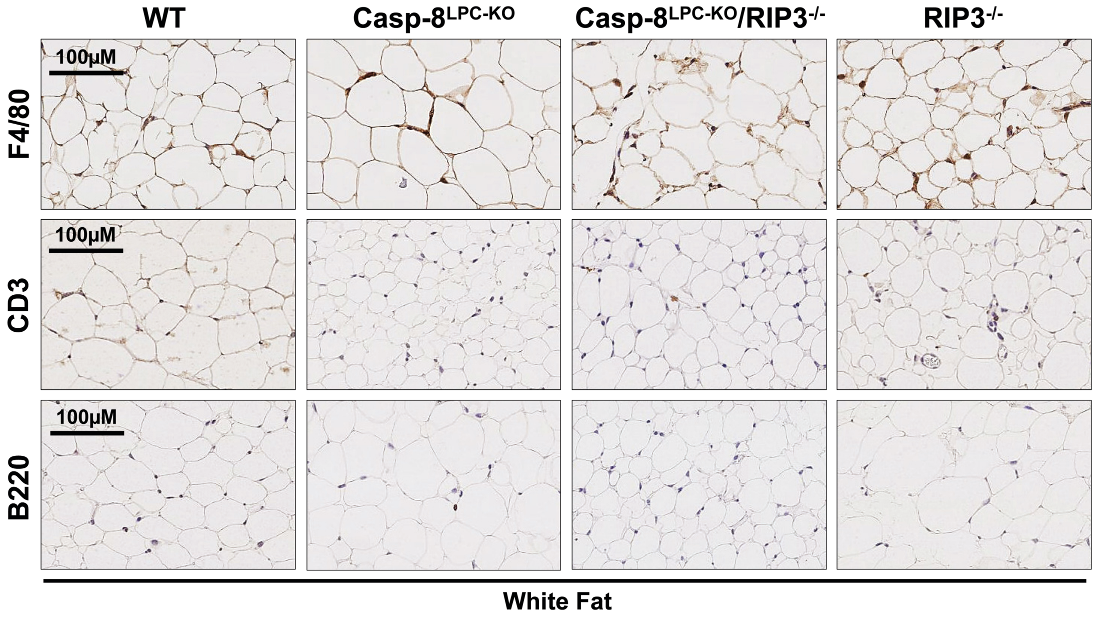

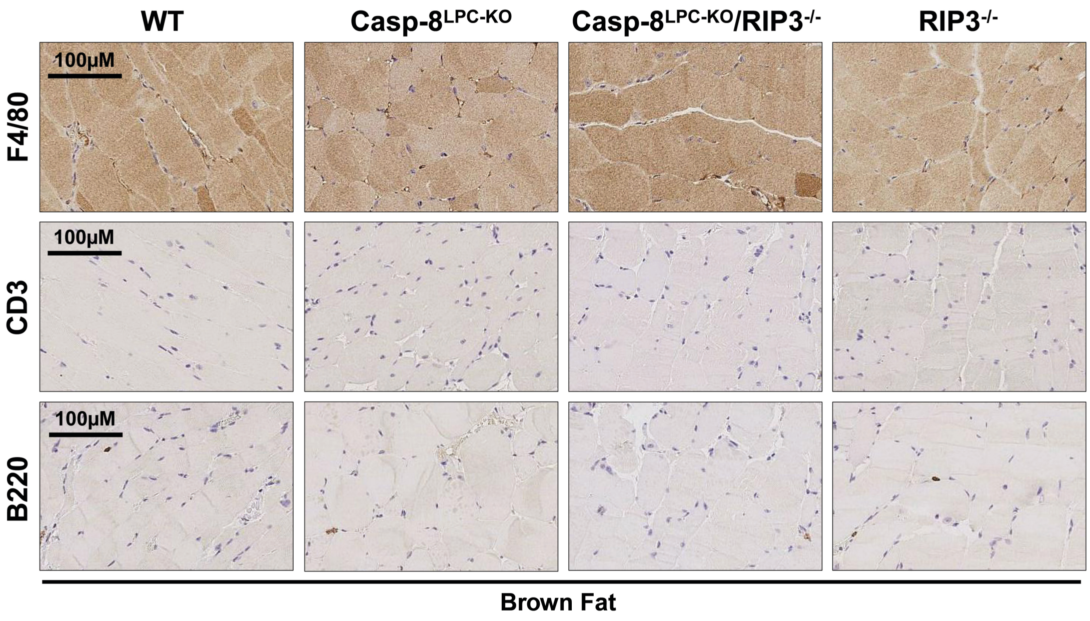


By responding point by point to all questions posed by the *Reviewers* and by performing additional work, aimed to address the questions of the *Reviewers* we now hope that our manuscript will be deemed appropriate for publication by EMBO Molecular Medicine. We would like to thank you for your enabling a careful, fair and swift review process and look forward to your response.

2nd Editorial Decision 19 May 2014

Thank you for the submission of your revised manuscript to EMBO Molecular Medicine. We have now received the enclosed reports from the Reviewers that were asked to re-assess it. As you will see the reviewers are now supportive and I am pleased to inform you that we will be able to accept your manuscript pending the following final amendments:

1) As per our Author Guidelines, the description of all reported data that includes statistical testing must state the name of the statistical test used to generate error bars and P values, the number (n) of independent experiments underlying each data point (not replicate measures of one sample), and the actual P value for each test (not merely 'significant' or 'P < 0.05').

2) Every published paper now includes a 'Synopsis' to further enhance discoverability. Synopses are displayed on the journal webpage and are freely accessible to all readers. They include a short standfirst - to be written by the editor - as well as 2-5 one-sentence bullet points that summarise the paper (to be written by the author). Please provide the short list of bullet points that summarise the key NEW findings. The bullet points should be designed to be complementary to the abstract - i.e. not repeat the same text. We encourage inclusion of key acronyms and quantitative information. Please use the passive voice. Please attach these in a separate file or send them by email, we will incorporate them accordingly.

3) Prof. Neumann's contribution appears to be missing from the Author Contributions section. Please complete.

4) We are now encouraging the publication of source data, particularly for electrophoretic gels and blots, with the aim of making primary data more accessible and transparent to the reader. Would you be willing to provide a PDF file per figure that contains the original, uncropped and unprocessed scans of all or at least the key gels used in the manuscript? The PDF files should be labeled with the appropriate figure/panel number, and should have molecular weight markers; further annotation may

be useful but is not essential. The PDF files will be published online with the article as supplementary "Source Data" files. If you have any questions regarding this just contact me.

5) In your point-by-point-response you included 2 figures. Would you please confirm whether or not we could publish this figure as part of the peer review process file?

I look forward to receiving your final revised version as soon as possible and in any case, possibly within two weeks

***** Reviewer's comments *****

Referee #1 (Remarks):

The authors have done a thorough job amending the study and bridging the original results and more limited model system to clinical samples of bona fide NASH patients. This is solid work which represents a clear advance to the field.

Referee #2 (Remarks):

Authors have done a number of additional studies to address the large numbers of requested edits by the reviewers. Although ultimately some of the requested items were not conducted (ie, studies in another model of NASH), the work is topical, interesting, and challenges the dogma which is good. Likely the mechanism of how caspase 8 functions in this disease (apoptosis vs necroptosis) probably varies in different models even of different models of NASH and accounts for the different results of different groups.

Referee #3 (Remarks):

Authors responded reviewer#3's comments satisfactorily.
